# Lipid metabolism: the potential therapeutic targets in glioblastoma

**DOI:** 10.1038/s41420-025-02390-3

**Published:** 2025-03-17

**Authors:** Lu Lu, Yan Zhang, Yuzhong Yang, Meihua Jin, Aiyu Ma, Xu Wang, Qiuyu Zhao, Xuemei Zhang, Jinhua Zheng, Xiang Zheng

**Affiliations:** 1 Department of Pathology, Affiliated Hospital of Guilin Medical University, Guilin, Guangxi China; 2 Department of Pathology, Liuzhou People’s Hospital Affiliated to Guangxi Medical University, Liuzhou, Guangxi China

**Keywords:** Cancer, Biomarkers

## Abstract

Glioblastoma is a highly malignant tumor of the central nervous system with a high mortality rate. The mechanisms driving glioblastoma onset and progression are complex, posing substantial challenges for developing precise therapeutic interventions to improve patient survival. Over a century ago, the discovery of the Warburg effect underscored the importance of abnormal glycolysis in tumors, marking a pivotal moment in cancer research. Subsequent studies have identified mitochondrial energy conversion as a fundamental driver of tumor growth. Recently, lipid metabolism has emerged as a critical factor in cancer cell survival, providing an alternative energy source. Research has shown that lipid metabolism is reprogrammed in glioblastoma, playing a vital role in shaping the biological behavior of tumor cells. In this review, we aim to elucidate the impact of lipid metabolism on glioblastoma tumorigenesis and explore potential therapeutic targets. Additionally, we provide insights into the regulatory mechanisms that govern lipid metabolism, emphasizing the critical roles of key genes and regulators involved in this essential metabolic process.

## Facts

- Despite advancements in treatment methods, the prognosis for patients diagnosed with glioblastoma remains poor.
- Accumulating evidence indicates that glioblastoma exhibits characteristics of dysregulated lipid metabolism.
- Recent studies have shown that drugs targeting key proteins and signaling pathways in lipid metabolism hold promise for glioblastoma therapy.

## Open questions

- How can we target tumor cells specifically while using targeted therapies for glioblastoma to minimize damage to normal cells?
- Systematic investigations are needed to explore the contributions of more specific types of lipid metabolism-associated molecules in driving glioblastoma tumorigenesis.
- Further research is required to develop safe lipid metabolism-targeting drugs with minimal side effects for glioblastoma treatment.

## Introduction

Glioblastoma is a highly aggressive malignancy of the central nervous system (CNS), classified as a grade 4 glioma. In WHO CNS5 classification, glioblastoma is referred to the astrocytic glioma characterized by isocitrate dehydrogenase (IDH)-wild type and H3-wild type and exhibits one or more features, including microvascular proliferation, necrosis, telomerase reverse transcriptase (TERT) promoter mutation, epidermal growth factor receptor (EGFR) gene amplification, and +7/−10 chromosome copy-number changes [1]. Glioblastoma accounts for 14.6% of all primary brain and other CNS tumors, 48.3% of primary malignant brain tumors, and 57.3% of all gliomas. The 5-year relative survival rate for glioblastoma patients is only 6.8%, with men being 1.58 times more likely to develop the disease compared to women [2]. Despite advancements in treatment methods, the prognosis for glioblastoma patients remains poor. Standard treatments, including surgery, radiotherapy, and chemotherapy, result in a median survival of only 12–15 months [3]. Genetic alterations in glioblastoma such as EGFR amplification are closely associated with tumor invasion and proliferation, presenting significant therapeutic targets. In recent years, many targeted therapies, such as EGFR/EGFRvIII antibodies and anti-EGFRvIII vaccinations, have been introduced. However, these interventions have shown limited effectiveness in improving patient prognosis in clinical trials [4]. The major drawbacks of current therapies primarily stem from high toxicity and drug resistance. The issue of toxicity largely arises from inadequate drug selectivity and non-specific targeting, which frequently result in off-target effects and systemic damage. Meanwhile, drug resistance arises through multifaceted mechanisms, primarily driven by tumor genetic instability and cellular heterogeneity. Additionally, sustained exposure to cytotoxic drugs can induce adaptive responses that foster therapeutic resistance. Key mechanisms underlying this phenomenon include: enzymatic inactivation, intracellular compartmentalization of therapeutic agents, modification of drug targets, and enhanced drug efflux via transporter proteins [5].

Lipids serve as a component of cell membranes, provide a crucial energy source for the human body, and play a number of roles in neurophysiological processes. Lipid messengers, such as endocannabinoids, prostaglandins, sphingosine-1-phosphate, and lysophosphatic acid, emerged as key regulators of neurodevelopment, synaptic plasticity, and inflammation [6]. In addition to hydrophilic neurotransmitters, such as glutamate and GABA, lipid messengers are synthesized on demand. The flux of lipid messengers is determined by the biosynthetic and metabolic rates of the involved enzymes, thereby controlling the magnitude and duration of their signaling and physiological response. When energy deficiency, β-fatty acid oxidation (FAO) can produce a large amount of ATP. Nicotinamide adenine dinucleotide (NADH) and flavin adenine dinucleotide (FADH2) generated by FAO can proceed to the electron transport chain to generate ATP via oxidative phosphorylation. Many cancers exhibit specific alterations in reprogramming lipid metabolism to maintain cell survival [7].

**Fig. 1:**
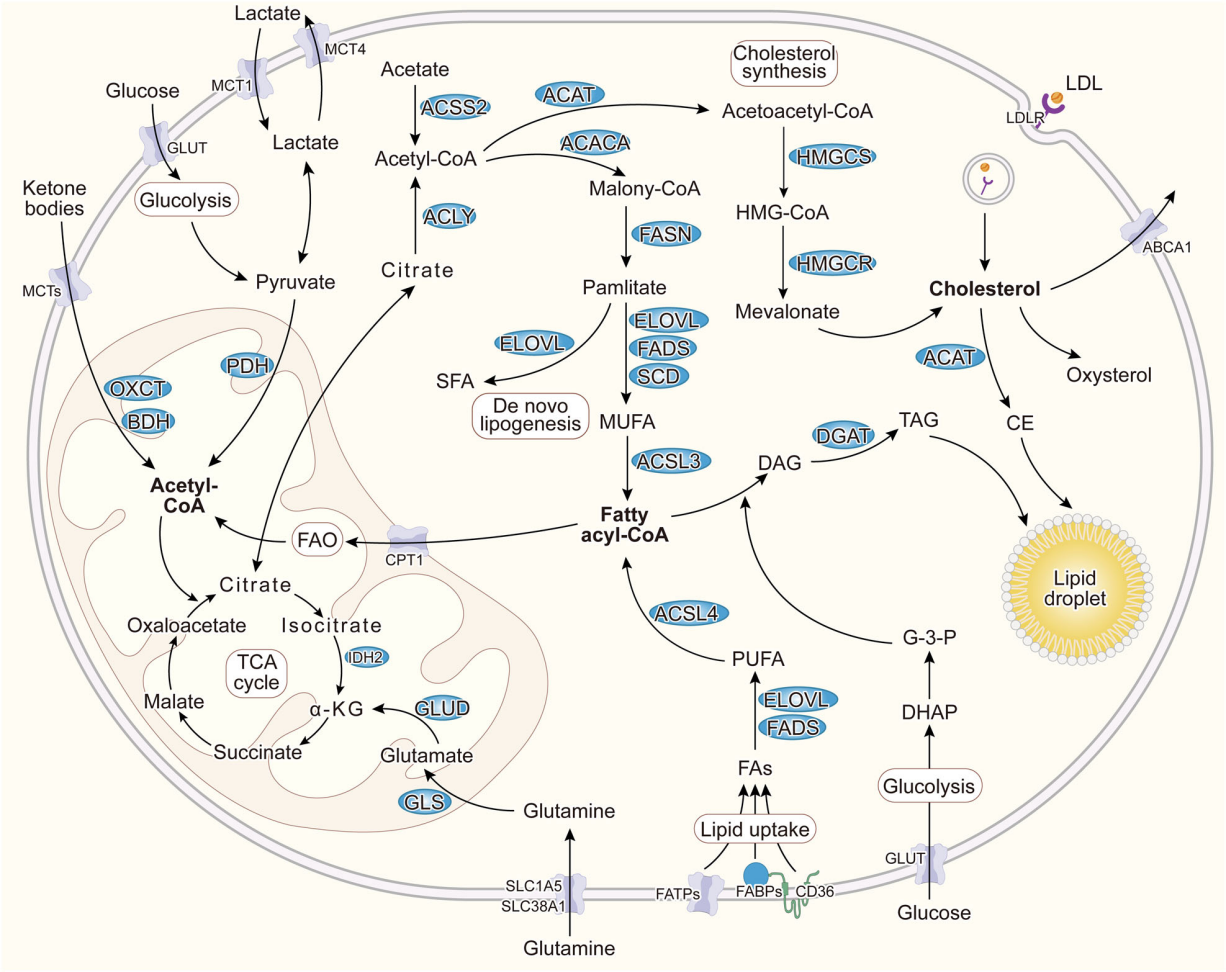
Lipid metabolism of tumor cells. Fatty acids from lipogenesis and uptake are catalyzed into acyl-CoA, facilitating FAO to generate acetyl-CoA, which then enters the TCA cycle to produce energy for tumor cell growth. Additionally, tumor cells can utilize glutamine, acetate, lactate, and ketone bodies to supply acetyl-CoA to the TCA cycle. Excess cholesterol, including acquired through uptake and synthesized de novo, is converted into CE. Surplus lipids are stored as LDs in the forms of CE and TAG, acting as cellular reserves. Under certain conditions, these lipid droplets undergo lipophagy and lipolysis to meet cellular energy demands.

Since the introduction of the Warburg effect, energy metabolism has emerged as a crucial factor in overcoming malignancies. The Warburg effect, proposed over a century ago, was the first indication of metabolic reprogramming in cancer, suggesting that the majority of tumor cells rely on aerobic glycolysis rather than oxidative phosphorylation for energy production. However, increasing evidence demonstrated that the efficiency of mitochondrial energy conversion is a critical metabolic factor of malignant tumor growth [8]. In tumor cells, energy metabolism can be reprogrammed to utilize conventional metabolic byproducts such as lactate, acetate, ketone bodies, and ammonia [9]. Notably, lipid metabolism exhibits heightened activity in tumor cells. This is characterized by increased uptake of exogenous lipids facilitated by upregulated lipid transporter proteins such as CD36, fatty acid transport protein (FATP), and fatty acid-binding protein (FABP). Additionally, tumor cells demonstrate versatility in citrate synthesis by utilizing glutamine, acetate, ketone bodies, and metabolic byproducts such as pyruvate from glycolysis and lactate to fuel acetyl-CoA production, thereby enhancing lipid biosynthesis. The conversion of fatty acids (FAs) into acyl-CoA, catalyzed by the long-chain acyl-coenzyme A synthetases (ACSLs), enables their mitochondrial entry through the pivotal enzyme carnitine palmitoyltransferase 1 (CPT1), facilitating FAO to generate acetyl-CoA, thus meeting cellular energy demands. Moreover, cholesterol is commonly transported to various tissues in the form of low-density lipoprotein. Tumor cells internalize cholesterol and its lipoproteins through the low-density lipoprotein receptor (LDLR) family expressed on their cell membranes. The surplus lipids are sequestered within lipid droplets (LDs) in the forms of cholesteryl esters (CE) and triacylglycerols (TAG) when energy supplies are high, serving as a reservoir for energy production via lipophagy and lipolysis in response to starvation (Fig. 1).

In glioblastoma cells, glycolysis is initially utilized as the primary means of energy metabolism. When this process is inhibited, glioblastoma cells shift to oxidative phosphorylation driven by elevated FAO to maintain energy production [10]. FAs transporters such as CD36, FABP, and FATP are overexpressed in glioblastoma, contributing to increased absorption and storage of lipids [11]. Research indicates that the expression of key enzymes in lipid metabolism is highly heterogeneous in glioblastoma cells, and the activity of these enzymes is reduced upon temozolomide (TMZ) treatment in newly diagnosed cases, but not in recurrent glioblastoma [12], which may reflect the tumor’s ability to adapt and develop resistance to TMZ by modifying the FA metabolism. Furthermore, disturbing the balance of cellular saturated and monounsaturated FA enhances TMZ toxicity in glioblastoma. This highlights a potential therapeutic strategy for treating recurrent glioblastoma. Additionally, lipid accumulation promotes angiogenesis and tumor-associated macrophage (TAM) infiltration in glioblastoma [13]. Further studies have revealed differences in lipid utilization and synthesis between TMZ-resistant and non-TMZ-resistant cells, with the former exhibiting higher levels of cholesterol and FAs and greater sensitivity to lipid synthase inhibitors [14]. Interestingly, the total lipid level in gliomas is lower than in normal controls [15], which may be related to the high level of lipid metabolism in tumor cells. These studies suggest the important role of lipid metabolism in glioblastoma. Investigating the lipid metabolism of glioblastoma represents a critical therapeutic strategy. Moreover, lipids have the potential to serve as diagnostic biomarkers for brain gliomas. Zhou et al. [16] used untargeted lipidomic analysis and identified a panel of 11 plasma lipids as candidate biomarkers in most malignant brain gliomas. Targeting the key metabolic enzymes and regulators involved in lipid metabolism, specifically those responsible for FA and cholesterol synthesis and uptake, provides a promising avenue to disrupt the metabolic cascade in glioblastoma and offers potential for therapeutic interventions for glioblastoma. Nanostructured carrier systems emerge as a promising platform for glioblastoma therapy, demonstrating drug stability and therapeutic efficacy. These advantages collectively enhance therapeutic performance while improving treatment safety. However, it is essential to recognize that these metabolic pathways are also crucial for normal cellular functions. Therefore, targeting lipid metabolism in glioblastoma therapy must carefully consider minimizing potential adverse effects on normal cells. In this review, we summarize the key proteins and signal pathways in lipid metabolism and their associated targeted drugs for glioblastoma therapy.

## Targets in fatty acid metabolism

FAO is recognized as a significant energy source for the development of cancer cells. Medium-chain fatty acids can serve as an alternative energy source capable of crossing the blood-brain barrier when brain glucose utilization is limited. FAs synthesis has been found to increase in decanoic (C10) acids stimulated glioblastoma cell U87MG [17]. Several studies have suggested that combining the AURKA inhibitor alisertib with the FAO inhibitor etomoxir reduces glioblastoma cell growth, enhances glioblastoma cell death, and extends overall survival in orthotopic glioblastoma patient-derived xenograft models [18]. Inhibition of FAO downregulates CD47 transcription and impairs tumor growth and immune evasion [19]. Additionally, a supplemented high-fat low-carbohydrate diet has been shown to slow tumor progression, increase survival, and reduce tumor burden in subcutaneous and orthotopic glioblastoma xenograft models [20]. These studies suggest that targeting FA metabolism in glioblastoma holds great promise for its treatment.

## Fatty acid synthase (FASN)

FASN is an enzyme that facilitates the synthesis of long-chain saturated FAs using acetyl-CoA and malonyl-CoA in the presence of NADPH. It is the primary multifunctional FA synthase in the human body, forming a homodimer with two identical functional chains, each containing eight functional structural domains. These domains are divided into three N-terminal domains (KS, MAT, and DH) and four C-terminal domains (ER, KR, ACP, TE), with a core region of 600 amino acids in the center of the protein, separating the N- and C-termini. The thioesterase (TE) functional domain, in particularly, determines the proportion of the final synthetic palmitic acid to other byproducts [21]. FASN is essential for brain function, and its expression in glioblastoma is elevated to meet the lipid requirements of tumor. FASN expression correlates with glioma grade and is found encapsulated in extracellular vesicles, potentially contributing to its dissemination in the bloodstream of glioblastoma patients [22]. Treating glioblastoma cells with FASN inhibitors, Orlistat, Cerulenin, and C75, induced autophagy and apoptosis, and reduced cell viability[23]. Additionally, FASN is involved in glioblastoma radio-resistance. Radiation increases FASN expression, leading to FAs and LDs accumulation, preventing ER stress, and inhibiting apoptosis. Inhibition of FASN significantly enhances the radiosensitivity of glioblastoma cells, and combining FASN blockade with focal radiation therapy prolongs the survival of glioblastoma-tumor-bearing mice [24]. Treating human glioma cells with Cerulenin to block FASN leads to an S-phase cell cycle block and induces apoptosis, while surprisingly, the viability of normal astrocytes remains unaffected [25]. Such selective action underscores the potential of Cerulenin as a compelling candidate for glioma-targeted therapy, minimizing potential adverse effects on normal cells and enhancing the overall safety profile of the treatment. However, reduced food intake and body weight were found in mice when treated with cerulenin and C75 [26], which potentially limits their therapeutic application for glioblastoma treatment. Among these inhibitors, Orlistat shows the most potent inhibitory effect in cell cultures by inducing autophagy and apoptosis and better security [23]. Additionally, low doses of Orlistat also inhibit pathological angiogenesis [27]. These studies suggest that FASN may be a target for glioblastoma. Nonetheless, it is crucial to evaluate the broader implications of using Orlistat, as its effects on normal cells and overall physiological processes must be carefully assessed to avoid detrimental impacts on healthy tissues and maintain systemic homeostasis during glioblastoma treatment.

## Acyl-CoA synthetase short-chain family member 2 (ACSS2)

ACSS2 converts acetate into acetyl-CoA. Under normal physiological conditions and with a high-quality diet, cells can increase lipid absorption by upregulation of ACSS2 and FABP1 expression. Research has shown that serum cholesterol, triglyceride, and phospholipid concentrations were significantly decreased in *Acss2*−/− mice fed a high-fat diet [28]. In tumor cells, ACSS2 expression can be upregulated to enhance acetate uptake, replacing the carbon supply for lipid metabolism under stressful conditions with hypoxia and low glucose supply [29, 30]. ACSS2 also converts butyrate into butyryl-CoA, which upregulates CPT1A activity, promotes FAO, and aids in the differentiation of iTreg cells, potentially helping malignancies evade the immune system [31]. Studies have shown that ACSS2 is overexpressed in glioblastoma, with a considerably higher concentration than in grade II and III gliomas. High concentrations of ACSS2 significantly increase the rate at which acetate is oxidized in the citric acid cycle, in order to meet the high biosynthetic and bioenergetic demands of malignant growth [32]. Notably, ACSS2 plays a pivotal role in glioblastoma development following its nuclear translocation. Phosphorylation of ACSS2 at S659 induces this translocation, where ACSS2 forms complexes with TFEB in the nuclei of glioblastoma cells. In the nucleus, ACSS2 locally produces acetyl-CoA for histone H3 acetylation in the promoter regions of lysosomal and autophagy-related genes, facilitating their upregulation, and promoting autophagy and cell survival under glucose deprivation conditions [33, 34]. Additionally, O-GlcNAc transferase (OGT) and O-GlcNAc levels are elevated in glioblastoma and are required for tumor growth in vitro and in vivo. OGT can activate ACSS2 following Ser-267 phosphorylation, and the regulation of glioblastoma cell growth in vivo is partly dependent on this phosphorylation. Interestingly, the O-GlcNAcase inhibitor promoted ACSS2 Ser-267 phosphorylation in glioblastoma cells, without affecting normal astrocytes [35], highlighting its potential for further application in glioblastoma therapy. ACSS2 inhibitors AD-5584 and AD-8007 significantly reduce lipid storage and acetyl-CoA levels and block colony cell survival and induce cell death in breast cancer brain metastasis cells in vitro, as well as inhibit tumor growth and survival in the brain microenvironment without causing overt toxicity to normal brain tissue [36]. Research by Gu et al. [37] found that hypoxia can upregulate ACSS2, which exerts a tumorigenic effect by regulating lipid metabolism through activation of the PI3K/AKT/mTOR pathway. Knockdown of ACSS2 and treatment with ACSS2 inhibitor have been shown to inhibit the progression of pancreatic neuroendocrine neoplasms. However, further research is needed to determine whether these findings are applicable to the treatment of glioblastoma.

## Fatty acid-binding protein 7 (FABP7)

FABP7, an intracellular FA chaperone, is involved in the uptake, transportation, metabolism, and storage of FAs [38]. Compared with glioblastoma fast-cycling cells, FABP7 is preferentially expressed in glioblastoma slow-cycling cells (SCCs). Inhibition of FABP7 by the inhibitor SB-FI-26 reduces lipid uptake and storage, which sensitizes glioblastoma SCCs to lower glucose levels and inhibition of migration [39]. Knockdown of FABP3 or FABP7 in glioblastoma cell line U87 reduces the formation of LDs during hypoxia, decreases cell number after hypoxia-reoxygenation, and impairs spheroid grown. Additionally, inhibition of FABP3 or FABP7 expression results in a significant delay in tumor growth and decreased lipid staining in U87 cells xenografts [40]. Irradiation at 2 Gy can increase the expression of FABP7 in glioblastoma-neutrosphere cell lines. Knockdown of FABP7 by specific siRNA reduces proliferation and migration in glioblastoma derived neurospheres GBMR11 NS and BT150 NS [41]. Compared to nuclear FABP7-negative, immunoreactivity of nuclear FABP7 in EGFR-expressing glioblastoma specimens correlates with poor survival. FABP7-specific antisense oligodeoxynucleotides inhibited EGF-induced migration in SF763 glioma cells [42]. Moreover, FABP7 deficiency has been suggested to decrease acetyl-CoA in astrocytes, suggesting that FABP7 affects acetyl-CoA generation [43]. This suggests glioblastoma with upregulated FABP7 may drive acetyl-CoA production to support lipid synthesis and rapid proliferation. Interestingly, lipids competing binding for FABP7 seem to lead to divergent migratory phenotypes. Mita et al. [44] found that ω-6 arachidonic acid (AA) promoted U87 migration, while ω-3 docosahexaenoic acid (DHA) suppressed migration in an FABP7-dependent manner. The uptake of DHA leads to inhibition of migration in glioblastoma neural stem-like cells A4-004N dependent on FABP7 expression, suggesting increasing DHA content may reduce glioblastoma migration in FABP7-expressing cells [45]. Umaru et al. found that among several FA treatments, oleic acid strongly promotes FABP7-mediated glioma cell proliferation [46]. The impact of FABP7-ligand interaction on glioblastoma cells may depend on the type of ligand and the microenvironment, suggesting that glioblastoma treatment targeting FABP7 should consider these factors.

## Targets in cholesterol metabolism

Survival of glioblastoma cells is known to depend on cholesterol [47]. In the body, the mevalonate pathway, governed by the key enzyme, is 3-hydroxy-3-methylglutaryl coenzyme-A-reductase (HMGCR), which is the primary route for endogenous cholesterol production. However, due to the impermeability of the blood-brain barrier, nearly all brain cholesterol is locally synthesized. In adults, neurons primarily rely on cholesterol delivery from neighboring cells such as astrocytes [48]. The brain-specific cholesterol metabolite 24S-hydroxycholesterol (24S-OHC) uniquely participates in the body’s cholesterol cycle, by crossing the blood-brain barrier [48]. Cholesterol plays a role in the biological behavior of glioblastoma. According to Bhat et al. [49], the combination of the dopamine receptor antagonist quetiapine and the HMGCR inhibitor atorvastatin, along with irradiation, significantly extended survival in mice bearing orthotopic patient-derived glioblastoma xenografts. However, Hao et al. [50] demonstrated increasing cholesterol concentration in glioblastoma cell membranes by avasimibe enhances T cell activation, exerting a significant anti-tumor effect. Therefore, strategically modulating cholesterol metabolism in glioblastoma holds promise for enhancing therapeutic outcomes. The subsequent sections will delve into the therapeutic implications of targeting cholesterol-related pathways for glioblastoma treatment.

## HMGCR

HMGCR is a rate-limiting enzyme in cholesterol synthesis that converts HMG-CoA into mevalonic acid (MVA). Studies have shown the upregulation of HMGCR in glioblastoma samples [51, 52]. A positive correlation between the expression of the multidrug resistance gene (MDR1) and HMGCR has been identified using data from the CGGA database [53]. Unlike normal brain cells, glioblastoma cells continue to express HMGCR despite high cell density, enabling them to continue using the mevalonate pathway for cholesterol synthesis at the expense of oxidative phosphorylation. This sustained expression of HMGCR in glioblastoma cells underscores its critical role in tumor metabolism and survival, making it a promising therapeutic target for glioblastoma treatment. Recent research has suggested HMGCR as a viable therapeutic target for glioblastoma treatment. Statins inhibit HMGCR, thereby blocking the conversion of HMG-CoA to MVA [54]. These compounds are widely used in clinical practice for cholesterol reduction, demonstrating their established safety profile and therapeutic potential. Statins have been shown to inhibit glioblastoma cell proliferation and induce apoptosis [55, 56]. Mechanistically, Statins inhibit HMGCR, leading to inhibition of geranylgeranyl pyrophosphate biosynthesis, a membrane anchoring molecule of Ras protein, ultimately inducing cell apoptosis by inhibiting the Ras/ERK and Ras/Akt pathways to increase activation of caspase-3 [56]. Similarly, Simvastatin has been found to attenuate tumor growth by activating the RAS-ERK pathway in brain tumor-initiating cells [52]. Furthermore, the administration of simvastatin with nanoparticles via the nose-to-brain route has experimentally demonstrated greater efficacy in inhibiting glioblastoma compared to intravenous injection [57]. This innovative delivery approach not only enhances the bioavailability of simvastatin in the CNS but also underscores the feasibility of repurposing statins as targeted drugs for glioblastoma, leveraging their pharmacological properties. Another research has demonstrated that HMGCR promoted glioblastoma growth and metastasis by enhancing TAZ expression, a critical mediator of the Hippo pathway [51]. However, a meta-analysis by Rendon et al. reviewed 64 publications showing that while statins inhibit glioma cell proliferation, migration, and invasion, their use is not associated with improved overall survival following glioblastoma surgery [58]. Surprisingly, an analysis of 483 gliomas, including 322 glioblastomas, from the Nurses’ Health Study, Nurses’ Health Study II, and the Male Health Professionals Follow-up Study revealed a significant association between statins use and increased glioma risk [59]. Therefore, further research is needed to explore the therapeutic potential of targeting HMGCR in glioblastoma.

## Oxysterol

Oxysterols are metabolites produced by the natural oxidation of cholesterol intracellularly or by enzymatic catalysis, which can act as endogenous activating ligands of the Liver X receptor (LXR) and participate in a variety of biological processes [60]. The effects of oxysterols on the body are primarily concentration-dependent. For instance, the particular brain steroid 24S-OHC at concentrations greater than 10 μM or 7-ketocholesterol (7KC) at concentrations greater than 30 μM drastically reduces the viability of human neuroblastoma cell SH-SY5Y, while 5 μM 24S-OHC shows protective effects against 7KC-induced cell death through transcriptional activation of the LXR signaling pathway [61]. 24S-OHC reduces cellular oxidative damage by increasing the cellular content of PGC-1α and TFAM in human glioblastoma U87MG cells at low concentrations (1 or 5 μM) [62].

Eibinger et al. [63] found that when glioblastoma cells are stimulated with IL-1β and TNFα, the synthesis of 25-OHC increased, which induces the chemotactic migration of THP-1 cells via the G protein-coupled receptor 183 (GPR183, also termed EBI2). This may recruit TAMs to glioblastoma tissues to modulate gliomagenesis. Interestingly, increasing the 25-OHC content in U87-MG cells reduces IL-1β level induced by lovastatin (LOVA) [64]. 27-OHC, another oxysterol, increases the expression of the cisplatin-resistance marker CD133 as well as proliferation, colony formation, epithelial-mesenchymal transition, migration, and invasion of glioblastoma cells. Moreover, 27-OHC is associated with shorter overall survival in glioblastoma patients [65]. Chronic exposure to 27-OHC results in increased tumorigenic and metastatic capacity and greater resistance to ferroptosis in cancer cells, which is reliant on the expression of the GPX4, a negative regulator of cellular ferroptosis [66]. In conclusion, oxysterols could be potential targets for the treatment of glioblastoma. However, it is important to note the effects of various oxysterol concentrations in cells and the phenomena of tumor cells adapting to oxysterol exposure.

## Targets in lipid droplets (LDs)

LDs are dynamic subcellular organelles found in most eukaryotic cells and a few prokaryotes, serving as a key mechanism for storing lipids to prevent toxicity from lipid peroxidation. LDs release lipids from the ER into the cytoplasm as outgrowths and are composed of an outer monolayer of phospholipids surrounding a core of neutral lipids. Triacylglycerol (TAG) and cholesterol esters are the essential components of LDs, with the amount of TAG directly determining LDs growth [67]. Additionally, over 200 proteins are responsible for regulating LDs activity, located in the outer monolayer of phospholipids. Some proteins, such as triglyceride-generating enzymes and cell death-inducing DFF45-like effector (CIDE) family proteins, control the size of typical LDs. LDs can undergo CIDE-mediated atypical fusion, representing a distinct mode of growth [67]. Tumor cells regulate LDs through multiple mechanisms. For instance, activating EGFR, overexpressing NRAS, or inhibiting PTEN can increase LDs production. This helps to prevent lipotoxicity and ER stress, enhances drug resistance and immune evasion, and provides lipid substrates and energy to fuel biological activity in tumor cells [68]. LDs are elevated in glioblastoma and inversely correlate with patient survival. Moreover, the number of LDs correlates with the Ki67 positive percentage in glioblastoma patients. Inhibition of sterol O-acyltransferase 1 (SOAT1) blocks LDs formation and suppresses glioblastoma growth via blocking the sterol regulatory element-binding protein 1 (SREBP-1)-regulated FA synthesis pathway [69]. Shakya et al. [70] found that intratumoral lipid metabolism heterogeneity exists, and LDs accumulate in the hypoxic core of glioblastoma organoids and also in perinecrotic and pseudopalisading regions of glioblastoma patient tumors. Bensaad et al. [40] found that hypoxia induces the accumulation of LDs in a HIF-1α-dependent manner, which is essential for glioblastoma cell growth and survival in the reoxygenation phase. Furthermore, energy deprivation caused by glucose scarcity in glioblastoma cells induces autophagy, releasing FAs from LDs into the cytoplasm for β-oxidation to provide cellular energy. Inhibition of autophagy or FAO under glucose-free conditions results in the accumulation of TAG/LDs and potentiates glioblastoma cell death [71]. Oxidizable PUFA sequestrated into LDs has been suggested to limit ferroptosis. CDKN2A deletion reduces oxidizable PUFA sequestration into LDs and sensitizes glioblastoma cells to ferroptosis [72]. Therefore, LDs play a crucial role in the malignant progression of glioblastoma, suggesting that limiting LDs utilization might be necessary for treating glioblastoma.

LDs are primarily utilized by cells through two processes: enzymatic hydrolysis mediated by lipases (lipolysis) and a selective form of autophagy (lipophagy) [73]. In glioblastoma cells, glucose deprivation induces choline kinase (CHK) α2 binding to LDs, mediated by AMPK-dependent CHKα2 S279 phosphorylation and KAT5-dependent CHKα2 K247 acetylation. This results in the phosphorylation of PLIN2/3, their dissociation from LDs, and subsequent degradation via Hsc70-mediated autophagy, ultimately leading to tumor growth. Elevated levels of CHKα2 S279 phosphorylation, CHKα2 K247 acetylation, and PLIN2/3 phosphorylation are associated with poor prognosis in glioblastoma patients [74]. The relationship between LDs and autophagy is intricate. While autophagy can degrade LDs, it can also lead to their formation [75]. Autophagy regulates the release of fat from cellular deposits like LDs and eliminates excess lipids to protect cells from lipotoxicity [76]. PARP inhibition downregulates the pro-survival AKT/mTOR pathway and induces the synthesis of LDs in glioblastoma cells. Autophagy and lipid turnover are involved in the resistance to PARP inhibition in these cells [77]. It is worth noting that the hydrophobic core of LDs can sequester lipophilic anticancer drugs, potentially reducing their effectiveness by preventing them from reaching their targets [5]. Zhang et al. demonstrated that curcumin’s lipophilic properties enable it to preferentially localize in lipid membranes and LDs. Inhibition of LDs accumulation using pyrrolidine-2 has been shown to effectively enhance the therapeutic efficacy of curcumin in glioblastoma [78]. Furthermore, the size of LDs significantly influences the efficacy of radiotherapy. Alkotub et al. [79] demonstrated that free Fenofibrate (FF) induces radiosensitivity in glioblastoma cells with small LDs by increasing DNA double-strand breaks. Conversely, large LDs can sequester free FF, thereby reducing its effectiveness and enhancing the radioprotective effect by limiting its availability. Their findings underscore the significance of investigating the heterogeneity of LDs in tumor cells to optimize therapeutic efficacy.

The enzyme SOAT1, also known as acyl-Coenzyme A: cholesterol acyltransferase 1 (ACAT1), primarily esterifies cholesterol in the ER and converts it to cholesterol esters that are then stored in LDs. Research reports that SOAT1 is upregulated in glioma tissues compared to normal brains, and high SOAT1 expression is associated with poor prognosis [80]. SOAT1 is mostly expressed in glioma-associated macrophages but less in glioblastoma cells [81]. SOAT1-mediated LDs formation plays a role in 24S-OHC-induced cell death, and knocking down SOAT1 can reduce 24S-OHC-induced cell death in human neuroblastoma SH-SY5Y cells [82]. Inhibition of SOAT1 decreases mitochondrial membrane potential, increases caspases 3/7 activity and p53 expression, and raises intracellular ROS levels in ovarian cancer cells [83]. Avasimibe, a SOAT inhibitor, increases glioblastoma sensitivity to ferroptosis and enhances the therapeutic effect of radiotherapy by increasing SLC40A1 expression [80]. Avasimibe inhibited cell growth by inducing cell cycle arrest and induced apoptosis through caspase-3 and caspase-8 activation in glioma cells [84]. Liu et al. [85] found that Avasimibe induced mitochondria-dependent apoptosis in glioblastoma cells by arresting the cell cycle at the G0/G1 and G2/M phases, which was mediated through the regulation of the p53/p21, p53/GADD45A, and Aurora A/PLK1 signaling pathways. Geng et al. [69] find that avasimibe suppresses glioblastoma growth via triggering feedback inhibition of SREBP and downstream lipogenesis enzymes ACC, FASN, and stearoyl-CoA desaturase-1 expression thus inhibiting FA synthesis. Mitotane, another SOAT1 inhibitor, is approved as an orphan drug for the treatment of adrenocortical carcinoma [86]. However, its treatment effects and adverse effects need further study in glioblastoma [81].

Similar to SOAT1, diacylglycerol o-acyltransferase 1 (DGAT1) also participates in the control of LDs. DGAT1 catalyzes the conversion of diacylglycerol (DAG) and acyl-CoA to TAG, which is then packaged into LDs. During autophagy, FAs are released and selectively channeled into new, clustered LDs by DGAT1. These new LDs are then decomposed by ATGL-mediated lipolysis, providing FAs to mitochondria for energy production. The DGAT1-dependent formation of LDs helps prevent acylcarnitine-induced mitochondrial dysfunction during starvation [87]. Thus, DGAT1-dependent sequestration of FAs as TAG in LDs protects mitochondria against lipotoxic and facilitates cell viability. Elevated DGAT1 expression, but not DGAT2, is associated with poorer overall survival among glioblastoma patients. Irradiation (IR) led to an increase in LDs which is decreased by DGAT1 knockdown. DGAT1 contributes radio-resistance by promoting triglyceride (TG) and LDs accumulation, preventing FAs from entering the mitochondria for FA oxidation [88]. Compared to normal brain tissue, DGAT1 is upregulated in glioblastoma tissues, and high DGAT1 expression is correlated with poor prognosis. Inhibiting DGAT1 by A-922500 alters lipid homeostasis and increases acylcarnitine levels, inducing mitochondrial damage and oxidative stress, and triggering apoptosis in glioblastoma cells. These results imply that targeting DGAT1 holds promise as a therapeutic avenue for glioblastoma [89]. DGAT inhibitors can prevent polyunsaturated fatty acids (PUFAs) accumulation as TGs in LDs and promote ferroptosis. Notably, the DGAT1 inhibitor A-922500 is more effective in preventing LDs formation compared to the DGAT2 inhibitor PF-06424439 [90]. Further evidence is needed to evaluate the effects of DGAT inhibitors in glioblastoma. In conclusion, inhibitors of SOAT1 and DGAT1 show promising efficacy in reducing LDs accumulation in glioblastoma cells. However, the specificity of these inhibitors and their potential effects on normal brain cells remain largely unexplored. Further investigation is essential to elucidate the dynamics and to assess the safety of these inhibitors in glioblastoma therapy.

## Targets in key regulators of lipid metabolism

Nuclear receptors (NRs), such as the Peroxisome Proliferator-Activated Receptors (PPARs), the Farnesoid X Receptor, and LXR, regulate genes by heterodimerizing with Retinoid X Receptor (RXR). These NRs have emerged as crucial regulators of lipid metabolism. Sterol regulatory element-binding protein 1 (SREBP-1), a master transcription factor in controlling FA synthesis, also plays a crucial role in lipid homeostasis. Dysregulation of lipid metabolism in glioblastoma is associated with several regulators, particularly PPARα, PPARγ, LXR, and SREBP-1.

**Fig. 2:**
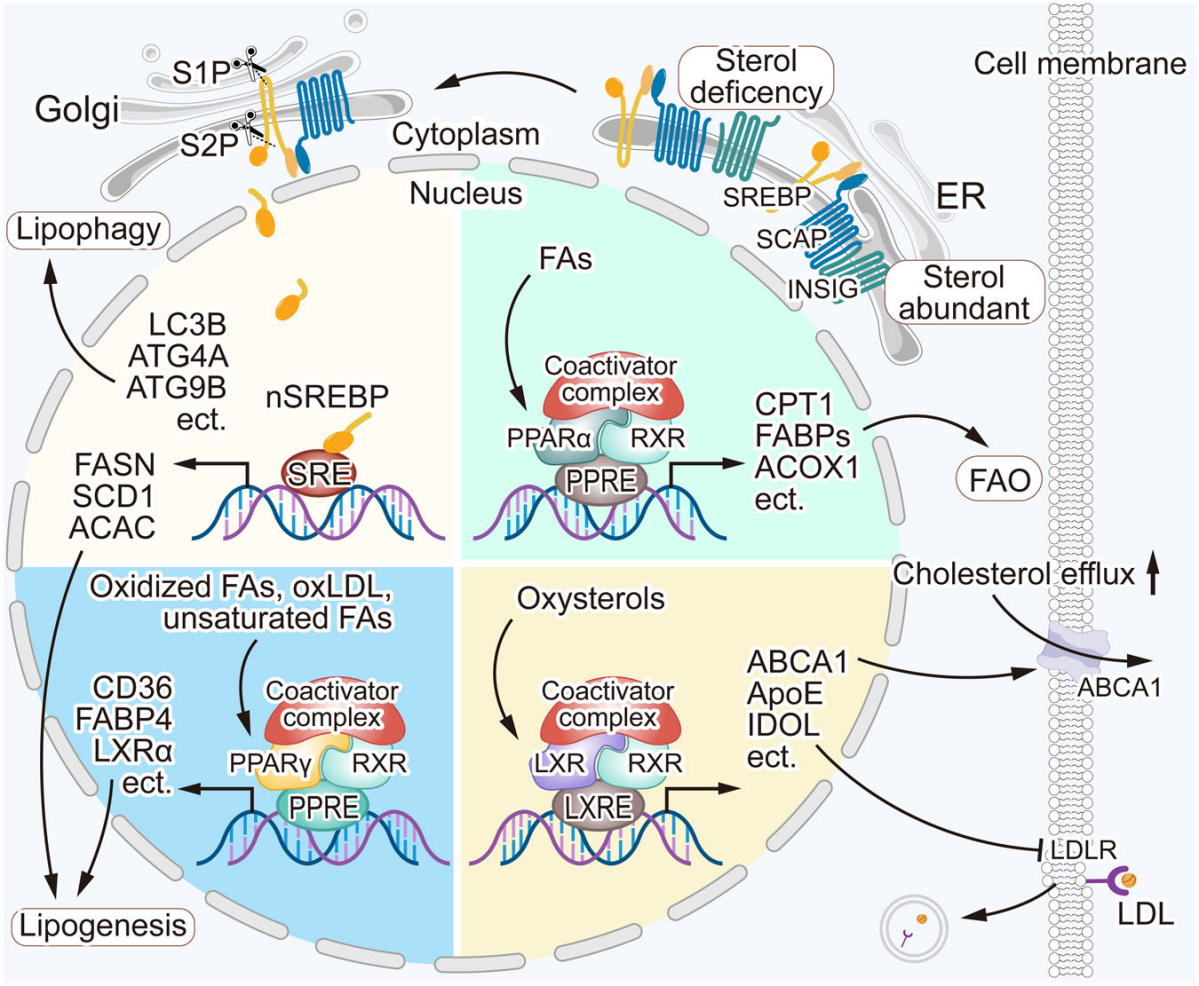
Regulation of lipid metabolism. Lipid metabolism is regulated by some key regulatory proteins. The regulatory mechanisms of PPARα, PPARγ, and LXRs are similar, as these receptors recruit coactivator proteins and form heterodimers with RXRs to activate lipid metabolism-associated proteins and maintain lipid homeostasis in the cells. SCAP acts as a sterol sensor, when sterol concentrations are high, sterols bind to SCAP, triggering SCAP and INSIG bind to each other, resulting in SREBPs, SCAP, and INSIG anchored in the ER. Conversely, when the sterol concentrations are low, the binding of SCAP and INSIG getting lost, and SCAP escorts SREBPs from the ER to the Golgi apparatus. There, the proteases S1P and S2P cleave SREBPs, releasing its N-terminal domain, which enters the nucleus and transcriptionally activates genes related to lipophagy and lipogenesis. The regulation of lipid metabolism is a complex and multifaceted process. Dysregulation of lipid metabolism regulatory mechanisms can contribute to glioblastoma tumorigenesis.

The mechanisms by which these proteins regulate lipid homeostasis are complex and influenced by various factors including FAs, cholesterol, and related derivatives (Fig. 2). After FAs enter into the nucleus, upon the adoption of them, PPARs recruit coactivator proteins and form heterodimers with RXRs to engage in target gene transcriptional activation by binding to the structural domain PPRE. PPARα targets genes related to lipid metabolism including CPT1, FABPs, ACOX1, ACSLs, etc. PPARα activation induces the expression of genes mainly involved in FAO, reduces FA and TG synthesis, and maintains energy homeostasis [91, 92]. PPARγ activation sensitizes insulin signaling, increases glucose utilization, and, compared to other isoforms, plays a more important role in adipogenesis and lipid synthesis [91, 93]. The mechanism of action of LXR is similar to that of PPAR but it is influenced by cholesterol and related derivatives such as oxysterols. LXR maintains intracellular cholesterol stabilization by regulating the expression of ABCA1, ApoE, IDOL, and other genes [94, 95]. SREBPs form a complex with SREBP cleavage-activating protein (SCAP), which acts as a sterol sensor. When sterol concentration is high, sterols bind to SCAP and alter its conformation, promoting its binding to INSIG, which results in SREBPs, SCAP, and INSIG being anchored in the ER. Conversely, when sterol concentration is low, SCAP escorts SREBPs in COPII vesicles from the ER to the Golgi apparatus. In the Golgi, two proteases, S1P and S2P, hydrolyze SREBPs, releasing their N-terminal structural domain, which then enters the nucleus to activate lipogenesis- and autophagy-related genes [96–98]. Several studies have shown that targeting these proteins may have therapeutic potential for glioblastoma.

## PPARα and PPARγ

PPARα, an intracellular FA receptor, plays a major role in regulating lipolysis. When FAs enter the cells, the PPARα and RXR complex activate specific DNA sequence components of PPREs to transcribe enzymes involved in FAO. In the brain, PPARα is activated by FAs synthesized by FASN, rather than from dietary fat. FASN-KO mice lack normal hypothalamic PPARα signaling, resulting in hypophagia [99]. PPARα is expected to serve as an important target for tumor immunotherapy. The complex tumor microenvironment of glioblastoma is influenced by PPARα, which affects tumor cell proliferation. Activated PPARα can boost the activity of regulatory T cells (Tregs), regulatory B cells (Bregs), and induce immune dysfunction of dendritic cells [92]. The PPARα antagonist AA452 downregulates the expression of c-Myc, cyclin D1, p-FAK, COX2, and pERK1/2 in glioblastoma primary cells, blocks cell proliferation, and increases sensitivity to radiotherapy [100]. MDM2-MDMX complex regulates lipids through altering PPARα activity by posttranslational modification. Lowered PPARα activity is essential for MDM2 and MDMX to promote ferroptosis [101]. Although these findings suggest that PPARα has a detrimental effect on promoting tumor growth, several reports indicate that PPARα can also inhibit glioblastoma growth. Fenofibrate, a PPARα ligand, can induce cell cycle arrest and suppress the growth of U87MG cells in a PPARα-dependent manner [102]. Another study suggested that the expression of PPARα protein and the PPARA gene is increased in glioblastoma samples, and glioblastoma patients with high PPARA expression had a significant increase in overall survival in the TCGA dataset [103].

PPARγ performs distinct functions from PPARα and mainly regulates lipid synthesis, resulting in increased lipid storage and improved insulin sensitivity and glucose metabolism through lipid-siphoning effects. However, PPARγ shares a comparable activation mechanism with PPARα [104]. PPARγ is highly expressed in the mesenchymal (MES) subtype glioblastoma. High PPARγ expression is linked to poor overall survival and disease-free survival of glioblastoma patients. PPARγ activation suppresses proneural-mesenchymal transition process by reducing the STAT3 signaling pathways and suppresses the growth and stemness of MES glioblastoma stem cells (GSCs) [105]. PPARγ also plays a role in controlling immune cell activation, which may contribute to tumor immune response. Cipolletta et al. [106] found that PPARγ is an important factor driving the accumulation and phenotype of Tregs residing in adipose tissue. PPARγ enhances the expression of Lrp8, Fabp5, Ldlr, and Scarb1 in lipid metabolism, providing CD4+ T cells with necessary FAs for early activation and proliferation [107]. Ercolano et al. [108] found that PPARγ plays a crucial role in supporting the IL-33-dependent ILC2s pro-tumorigenic functions. The regulation of lipid metabolism by both PPARα and PPARγ plays a crucial role in shaping the biological behavior of glioblastoma, which is involved in lipid homeostasis and cellular signaling, and presents promising avenues for therapeutic intervention in glioblastoma.

## LXRs

LXRs are classified into LXRα and LXRβ isoforms, which regulate lipoproteins in cells to maintain intracellular cholesterol stability. The regulation of LXRs is becoming increasingly significant in the field of glioblastoma treatment. In glioblastoma cells, the dysregulation of cholesterol regulatory and surveillance mechanisms leads to the accumulation of intracellular cholesterol. Interestingly, glioblastoma cells show higher sensitivity to exogenous LXR ligands than normal astrocytes. Over-activation of LXRs in glioblastoma reduces cellular cholesterol and induces tumor cell death, whereas astrocytes remain unaffected. This differential sensitivity may be due to the decreased capacity of glioblastoma cells to produce endogenous LXR ligands, such as oxysterols [47]. Notably, research has discovered that, in addition to enhancing cholesterol efflux, LXRs agonists can impair TCA cycle and oxidative phosphorylation (OXPHOS) in tumor cells, promote the expression of the Bcl2 apoptotic family protein Noxa, and enhance apoptosis [109]. GW3965 and LXR-623, the two most frequently utilized LXR agonists, have shown potential in the treatment of glioblastoma [47, 110]. Additionally, elevating ApoE via LXR activation suppresses the survival of myeloid-derived suppressor cells (MDSC) through its action on LRP8 receptors, and increases the number of cytotoxic T lymphocytes [111]. Patel et al. [112] found that LXRβ signaling modulates cholesterol homeostasis and enables continued proliferation and viability under conditions of high density in glioma cells. LXRβ gene expression correlates with poor patient prognosis with the classical glioblastoma subtype, suggesting targeted LXRβ might be effective in the treatment of glioblastoma. Collectively, targeting LXR signaling has shown potential in the therapy of glioblastoma, with agonists GW3965 and LXR-623 as promising candidates for therapeutic intervention.

## SCAP and SREBP-1

SREBPs play a role in intracellular nutrient-energy metabolism, with significant implications for tumor growth. SCAP, a critical sterol sensor, is essential for initiating SREBP activation [113, 114]. There are three isoforms of SREBPs: SREBP-1a, SREBP-1c, and SREBP-2. SREBP-1a is able to induce both lipogenic and cholesterol gene expression, SREBP-1c is primarily responsible for lipogenic gene expression, and SREBP-2 mainly regulates cholesterol metabolism [115]. In conditions of cholesterol deficiency in glioblastoma, activated SREBP-1 promotes LDs lipophagy by upregulating critical autophagic genes, leading to the hydrolysis of esterified cholesterol and thus maintaining plasma membrane cholesterol homeostasis [97]. Research by Cheng et al. [116] found that EGFR signaling enhances SCAP and its N-glycosylation by increasing glucose uptake, which subsequently activates SREBP-1 in glioblastoma cells. This suggests that SCAP N-glycosylation is a critical factor for EGFRvIII-induced glioblastoma tumorigenesis. In glioblastoma cells, EGFR-PI3K-Akt signaling promotes SREBP-1 cleavage and increases FA concentration. Knockdown of SREBP-1 promotes the death of EGFRvIII-bearing glioblastoma [117]. These findings suggest that targeting SREBP-1 and EGFR may be a promised therapeutic approach for treating EGFR-activated glioblastoma. In TME, upregulated SREBP activity and PD-1 expression are observed in Treg cells. SCAP/SREBP signaling promotes PD-1 expression in Treg cells, and specific deletion of SCAP of Treg cells leads to an inhibition of tumor growth and an increased effectiveness of anti-PD-1 immunotherapy in mice [118]. This indicates that SCAP/SREBP plays an important role in the TME. SREBP-1 also interacts with FBI-1, a proto-oncogene, directly via their DNA binding domains, synergistically enhancing the transcriptional activation of the SREBP-responsive promoter and FASN genes [119].

PF-429242, Betulin, and Fatostatin are prominent inhibitors of the SREBP pathway. Among these, only Fatostatin has exhibited antimitotic properties. Given Fatostatin’s ability to inhibit SREBP activity and impeding cell proliferation, invasion, and migration, it shows potential for application in tumors with elevated lipid metabolism and rapid proliferation rates, such as glioblastoma [120, 121]. Recent studies have identified additional compounds with potential as novel therapeutic agents for treating glioblastoma by inhibiting SREBP activity. Lycorine, for instance, has been shown to exert in vitro anti-tumor activity, including inhibition of growth and migration in U373 glioblastoma cells [122]. Lycorine accelerates the degradation of SCAP through the SQSTM1-mediated autophagy-independent lysosomal degradation pathway, binds to SCAP, inhibits SREBFs activity, and downregulates both cholesterol and FA synthesis [123]. Berberine (BBR) modulated lipogenesis through inhibiting SREBP-1 activation, resulting in the inhibition of colon cancer cell proliferation [124]. In glioblastoma, BBR has been found to induce high autophagy flux and reduce glycolytic capacity. It inhibits cell proliferation, migration, and invasion, and induces autophagy in glioblastoma cells [125]. BBR-Loaded nanoparticles have been shown to induce cytotoxicity events in the T98G glioblastoma cell line, with a further increase following the photodynamic stimulation. BBR-nanoparticles-based strategy coupled with photoactivation suggests a promising therapeutic strategy for glioblastoma [126]. Importantly, nanoparticles of BBR have no significant cytotoxicity on normal rat primary astrocytes, which might reduce potential side effects. This method holds promise for improving treatment outcomes in glioblastoma patients without compromising normal brain function. Glioblastoma cell lines U87MG, A172, and T98G treated with phytol (PHY) at IC50 significantly reduced SREBP-1 expression and cell viability [127]. However, it should be noted that SCAP deletion in astrocytes causes maturation impairment of the hippocampal spine [128]. Although inhibiting SREBP activity seems to be a feasible therapeutic strategy for glioblastoma, further researches are needed to resolve the specificity in glioblastoma.

## Targets in N6-methyladenosine (m6A) machinery

The m6A modification, an epigenetic methylation of the N atom at position 6 of the adenine base A, is currently considered the most prevalent, abundant, and conserved internal modification of RNA. It is mainly regulated by three types of proteins: methyltransferases (writers, including METTL3, METTL14, WTAP, etc.), demethylases (erasers, including FTO and ALKBH5), and m6A-binding proteins (readers). The m6A machinery naturally participates in the regulation of lipid metabolism. For instance, hepatocyte-specific knockout of Mettl3 suppresses the expression of lipid metabolism-related gene expression, including Adh7, Cpt1a, and Cyp7a1, contributing to lipid metabolism disorders [129]. Specific deletion of Fto in lipid reduced brain lipid level and impaires the cognitive function of mice. Fto deficiency leads to adenosine accumulation, resulting in the apoptosis of adult neural stem cells [130]. WTAP enhances AR methylation and suppresses its expression in a YTHDF2-dependent manner, promoting mitochondrial lipid oxidation [131]. m6A modification mediated by METTL14 has been found to elevate the expression of *circRNA_103239*, resulting in the inhibition of glioma progression [132]. hnRNPA2B1, an m6A reader, stabilizes m6A-modified SREBP-2 mRNA and triggers de novo cholesterol synthesis, contributing to glioma stemness and malignancy [133].

In glioblastoma tissue, m6A machinery proteins, including METTL3, METTL14, WTAP, FTO, ALKBH5, YTHDF1, YTHDF2, YTHDF3, HNRNPC, etc. are significantly upregulated compared to normal brain tissue [134–136]. Upregulated WTAP contributes to a high ROS environment that promotes the malignant progression of glioblastoma cells [135]. The expression of ALKBH5 is positively correlated with glioma malignant phenotypes, suggesting its potential as a promising prognostic factor for glioblastoma patients. Downregulation of ALKBH5 in glioblastoma cells inhibits the expression of vascular endothelial growth factor A (VEGFA) and impairs the angiogenic potential of co-cultured HUVECs [136]. Interestingly, Lv et al. [137] found that EGF-induced Y71 phosphorylation of ALKBH5 is essential for its nuclear export. ALKBH5 promoted GSCs’ survival from ferroptosis through increasing glutamate-cysteine ligase modifier subunit mRNA by impeding YTHDF2-mediated decay. Pharmacologic targeting of ALKBH5 enhanced the anti-tumor efficacy of EGFR inhibitor erlotinib, suggesting that ALKBH5 may function as a potential therapeutic target in combination with EGFR for glioblastoma patients. In conclusion, the potential use of m6A machinery proteins as therapeutic targets for the treatment of glioblastoma appears promising. Nevertheless, further research is required to fully understand the specific regulatory mechanisms of m6A in glioblastoma to optimize therapeutic strategies.

## Perspectives

**Fig. 3:**
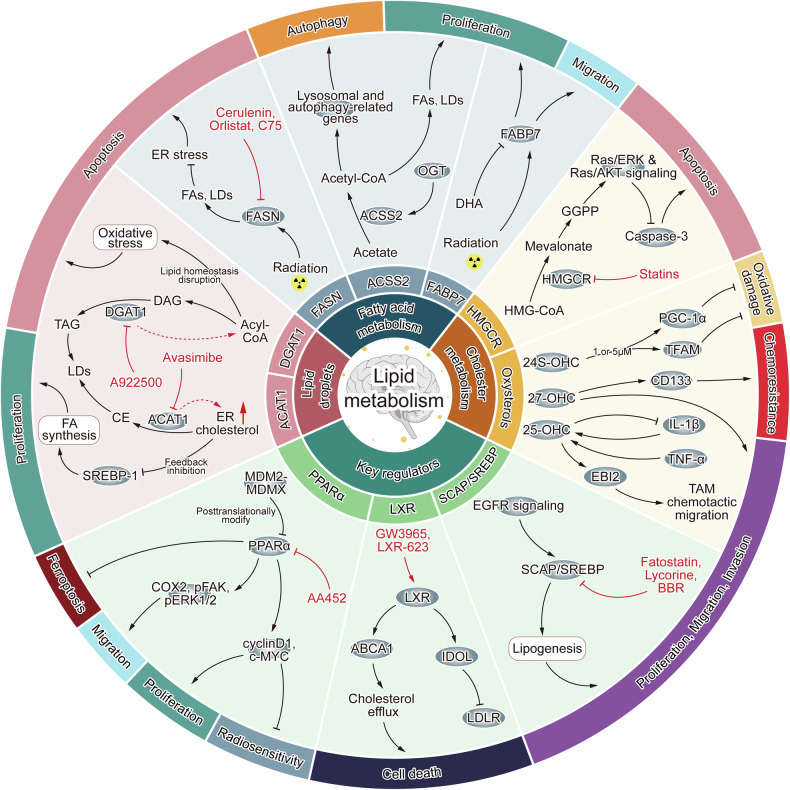
Lipid metabolism influences the biological behavior of glioblastoma. Fatty acid metabolism, cholesterol metabolism, LDs, and key regulatory proteins impact glioblastoma through multiple pathways, affecting processes such as tumor cell proliferation, migration, invasion, apoptosis, autophagy, ferroptosis, radiosensitivity, and chemoresistance. Certain inhibitors and agonists can influence tumor biological behavior by modulating lipid metabolism in glioblastoma. Examples include FASN inhibitors such as Cerulenin, C75, and Orlistat; HMGCR inhibitors such as statins; SREBP-SCAP pathway inhibitors such as Fatostatin, Lycorine, and Berberine (BBR); the PPARα inhibitor AA452; the DGAT1 inhibitor A-922500; the ACAT1 inhibitor Avasimibe; and the LXR agonists GW3965 and LXR-623.

Darwish et al. [138] summarized how the products of lipid metabolism impacted glioblastoma progression and outlined some clinical trials of drugs that targeted lipid metabolism in glioblastoma. Yu et al. [139] conducted a comprehensive review of serum lipidomics applications in glioblastoma diagnosis, prognosis, and therapeutic target. Miska’s team [140] summarized the metabolic fates of FAs in glioblastoma, encompassing anabolism, catabolism, regulation of ferroptosis, and the production of signaling molecules, and showed the strategies to perturb these pathways. Furthermore, Kou et al. [141] reviewed the multifaceted regulatory networks of lipid metabolism in glioblastoma and the roles of LDs in tumor development. In this review, we systematically summarized how lipid metabolism influenced various aspects of glioblastoma cell behavior, including proliferation, migration, invasion, apoptosis, autophagy, ferroptosis, radiosensitivity, and chemoresistance, and discussed the application prospect of targeted inhibitors in treatment of glioblastoma (Fig. 3). Drugs that target lipid metabolic pathways such as synthesis, decomposition, LDs formation and regulation are currently being developed for glioblastoma treatment (Table 1). Although several inhibitors and agonists targeting lipid metabolism have shown potential therapeutic effects in glioblastoma, further research is needed, including evaluation of combination therapy and adverse reactions. Resolving the structures of key proteins and enzymes in lipid metabolism will accelerate the discovery of new potent inhibitors for glioblastoma therapy. Combining radiotherapy, chemotherapy, and targeted therapies aimed at modulating lipid metabolism has demonstrated considerable anti-tumor efficacy in glioblastoma, underscoring the importance of targeting lipid metabolism as a therapeutic strategy. However, the high degree of tumor heterogeneity and metabolic adaptability that glioblastoma cells exhibit in response to environmental stimuli and energy requirements pose significant challenges. Drugs and inhibitors that target lipid metabolism may not only affect tumor cells but also normal cells, leading to potential toxicity and adverse effects. Therefore, increasing the therapeutic selectivity towards tumor cells while minimizing toxicity to normal cells remains a significant challenge. It is crucial to emphasize that the combination of emerging nanotechnology with pitavastatin or TMZ, has significantly enhanced the therapeutic efficacy of these drugs in the context of glioblastoma treatment [142, 143]. The application of nanostructured carriers presents high drug stability and effectiveness, which improves product quality, anticancer efficacy, and patient safety. Consequently, the nanostructured carrier system stands as a promising anticancer platform for the therapy of glioblastoma. It is crucial to consider the complex interplay among patient characteristics, tumor tissue features, and metabolic processes during practical application. Further research is necessary to fully elucidate the intricate mechanisms involved in the lipid metabolism process in glioblastoma. This is essential for developing more effective and safer therapeutic strategies for glioblastoma, ultimately improving patient outcomes.

**Table 1:** Inhibitors of potential therapeutic targets in lipid metabolism in glioblastoma.

Inhibitors	Targets	Functions	References
Orlistat, Cerulenin and C75	FASN	Induction of apoptosis and autophagy	[23]
Cerulenin	FASN	Induction of apoptosis and S-phase cell cycle arrest	[25]
SB‐FI‐26	FABP7	Inhibition of glioblastoma slow‐cycling cell migration	[39]
Statin	HMGCR	Inhibition of cell proliferation and induction of autophagy	[55]
		Induction of apoptosis	[56]
Avasimibe	ACAT1	Enhancement of ferroptosis sensitivity	[80]
		Inhibition of cell proliferation and induction of apoptosis	[84, 85]
A-922500	DGAT1	Induction of apoptosis	[89]
AA452	PPARα	Induction of cell death, enhancement of radiosensitivity, and inhibition of migration	[100]
Fatostatin	SCAP	Inhibition of cell proliferation	[120]
Lycorine	SCAP	Inhibition of cell proliferation and migration	[122]
Berberine	SREBP-1	Inhibition of cell proliferation, migration and invasion	[125]
Phytol	SREBP-1	Inhibition of cell viability	[127]

## References

[1] McNamara C, Mankad K, Thust S, Dixon L, Limback-Stanic C, D’Arco F, et al. 2021 WHO classification of tumours of the central nervous system: a review for the neuroradiologist. Neuroradiology. 2022;64:1919–50. 35869291 10.1007/s00234-022-03008-6

[2] Ostrom QT, Cioffi G, Gittleman H, Patil N, Waite K, Kruchko C, et al. CBTRUS Statistical Report: primary brain and other central nervous system tumors diagnosed in the United States in 2012–2016. Neuro Oncol. 2019;21:v1–100. 31675094 10.1093/neuonc/noz150 PMC6823730

[3] Cloughesy TF, Cavenee WK, Mischel PS. Glioblastoma: from molecular pathology to targeted treatment. Annu Rev Pathol. 2014;9:1–25. 23937436 10.1146/annurev-pathol-011110-130324

[4] An Z, Aksoy O, Zheng T, Fan QW, Weiss WA. Epidermal growth factor receptor and EGFRvIII in glioblastoma: signaling pathways and targeted therapies. Oncogene. 2018;37:1561–75. 29321659 10.1038/s41388-017-0045-7 PMC5860944

[5] Antunes P, Cruz A, Barbosa J, Bonifácio VDB, Pinto SN. Lipid droplets in cancer: from composition and role to imaging and therapeutics. Molecules. 2022;27:991. 35164256 10.3390/molecules27030991 PMC8840564

[6] Punt JM, van der Vliet D, van der Stelt M. Chemical probes to control and visualize lipid metabolism in the brain. Acc Chem Res. 2022;55:3205–17. 36283077 10.1021/acs.accounts.2c00521 PMC9670861

[7] Santos CR, Schulze A. Lipid metabolism in cancer. FEBS J. 2012;279:2610–23. 22621751 10.1111/j.1742-4658.2012.08644.x

[8] Potter M, Newport E, Morten KJ. The Warburg effect: 80 years on. Biochem Soc Trans. 2016;44:1499–505. 27911732 10.1042/BST20160094 PMC5095922

[9] Sun L, Suo C, Li ST, Zhang H, Gao P. Metabolic reprogramming for cancer cells and their microenvironment: beyond the Warburg Effect. Biochim Biophys Acta Rev Cancer. 2018;1870:51–66. 29959989 10.1016/j.bbcan.2018.06.005

[10] Nguyen TTT, Zhang Y, Shang E, Shu C, Torrini C, Zhao J, et al. HDAC inhibitors elicit metabolic reprogramming by targeting super-enhancers in glioblastoma models. J Clin Investig. 2020;130:3699–716. 32315286 10.1172/JCI129049 PMC7324177

[11] Tamas C, Tamas F, Kovecsi A, Cehan A, Balasa A. Metabolic contrasts: fatty acid oxidation and ketone bodies in healthy brains vs. glioblastoma multiforme. Int J Mol Sci. 2024;25:5482. 38791520 10.3390/ijms25105482 PMC11122426

[12] Parik S, Fernandez-Garcia J, Lodi F, De Vlaminck K, Derweduwe M, De Vleeschouwer S, et al. GBM tumors are heterogeneous in their fatty acid metabolism and modulating fatty acid metabolism sensitizes cancer cells derived from recurring GBM tumors to temozolomide. Front Oncol. 2022;12:988872. 36338708 10.3389/fonc.2022.988872 PMC9635944

[13] Rohrig F, Schulze A. The multifaceted roles of fatty acid synthesis in cancer. Nat Rev Cancer. 2016;16:732–49. 27658529 10.1038/nrc.2016.89

[14] Choo M, Mai V-H, Kim HS, Kim D-H, Ku J-L, Lee SK, et al. Involvement of cell shape and lipid metabolism in glioblastoma resistance to temozolomide. Acta Pharmacol Sinica. 2022;44:670–9. 10.1038/s41401-022-00984-6 PMC9958008 36100765

[15] Kökoğlu E, Görseval A, Sönmez H, Ozyurt E. Tissue lipid composition of human gliomas and meningiomas. Cancer Lett. 1992;65:169–71. 1511422 10.1016/0304-3835(92)90162-o

[16] Zhou J, Ji N, Wang G, Zhang Y, Song H, Yuan Y, et al. Metabolic detection of malignant brain gliomas through plasma lipidomic analysis and support vector machine-based machine learning. EBioMedicine. 2022;81:104097. 35687958 10.1016/j.ebiom.2022.104097 PMC9189781

[17] Damiano F, De Benedetto GE, Longo S, Giannotti L, Fico D, Siculella L, et al. Decanoic acid and not octanoic acid stimulates fatty acid synthesis in U87MG glioblastoma cells: a metabolomics study. Front Neurosci. 2020;14:783. 32792906 10.3389/fnins.2020.00783 PMC7390945

[18] Nguyen TTT, Shang E, Shu C, Kim S, Mela A, Humala N, et al. Aurora kinase A inhibition reverses the Warburg effect and elicits unique metabolic vulnerabilities in glioblastoma. Nat Commun. 2021;12:5203. 34471141 10.1038/s41467-021-25501-x PMC8410792

[19] Jiang N, Xie B, Xiao W, Fan M, Xu S, Duan Y, et al. Fatty acid oxidation fuels glioblastoma radioresistance with CD47-mediated immune evasion. Nat Commun. 2022;13:1511. 35314680 10.1038/s41467-022-29137-3 PMC8938495

[20] Martuscello RT, Vedam-Mai V, McCarthy DJ, Schmoll ME, Jundi MA, Louviere CD, et al. A supplemented high-fat low-carbohydrate diet for the treatment of glioblastoma. Clin Cancer Res. 2016;22:2482–95. 26631612 10.1158/1078-0432.CCR-15-0916

[21] Mullen GE, Yet L. Progress in the development of fatty acid synthase inhibitors as anticancer targets. Bioorg Med Chem Lett. 2015;25:4363–9. 26364942 10.1016/j.bmcl.2015.08.087

[22] Ricklefs FL, Maire CL, Matschke J, Duhrsen L, Sauvigny T, Holz M, et al. FASN is a biomarker enriched in malignant glioma-derived extracellular vesicles. Int J Mol Sci. 2020;21:1931. 32178271 10.3390/ijms21061931 PMC7139767

[23] Grube S, Dünisch P, Freitag D, Klausnitzer M, Sakr Y, Walter J, et al. Overexpression of fatty acid synthase in human gliomas correlates with the WHO tumor grade and inhibition with Orlistat reduces cell viability and triggers apoptosis. J Neurooncol. 2014;118:277–87. 24789255 10.1007/s11060-014-1452-z

[24] De Martino M, Daviaud C, Minns HE, Lazarian A, Wacker A, Costa AP, et al. Radiation therapy promotes unsaturated fatty acids to maintain survival of glioblastoma. Cancer Lett. 2023;570:216329. 37499741 10.1016/j.canlet.2023.216329

[25] Zhao W, Kridel S, Thorburn A, Kooshki M, Little J, Hebbar S, et al. Fatty acid synthase: a novel target for antiglioma therapy. Br J Cancer. 2006;95:869–78. 16969344 10.1038/sj.bjc.6603350 PMC2360524

[26] Loftus TM, Jaworsky DE, Frehywot GL, Townsend CA, Ronnett GV, Lane MD, et al. Reduced food intake and body weight in mice treated with fatty acid synthase inhibitors. Science. 2000;288:2379–81. 10875926 10.1126/science.288.5475.2379

[27] Bruning U, Morales-Rodriguez F, Kalucka J, Goveia J, Taverna F, Queiroz KCS, et al. Impairment of Angiogenesis by Fatty Acid Synthase Inhibition Involves mTOR Malonylation. Cell Metab. 2018;28:866–80.e15. 30146486 10.1016/j.cmet.2018.07.019 PMC8057116

[28] Huang Z, Zhang M, Plec AA, Estill SJ, Cai L, Repa JJ, et al. ACSS2 promotes systemic fat storage and utilization through selective regulation of genes involved in lipid metabolism. Proc Natl Acad Sci USA. 2018;115:E9499–506. 30228117 10.1073/pnas.1806635115 PMC6176566

[29] Munir R, Lisec J, Swinnen JV, Zaidi N. Lipid metabolism in cancer cells under metabolic stress. Br J Cancer. 2019;120:1090–8. 31092908 10.1038/s41416-019-0451-4 PMC6738079

[30] Yoshii Y, Waki A, Furukawa T, Kiyono Y, Mori T, Yoshii H, et al. Tumor uptake of radiolabeled acetate reflects the expression of cytosolic acetyl-CoA synthetase: implications for the mechanism of acetate PET. Nucl Med Biol. 2009;36:771–7. 19720289 10.1016/j.nucmedbio.2009.05.006

[31] Hao F, Tian M, Zhang X, Jin X, Jiang Y, Sun X, et al. Butyrate enhances CPT1A activity to promote fatty acid oxidation and iTreg differentiation. Proc Natl Acad Sci USA. 2021;118:e2014681118. 34035164 10.1073/pnas.2014681118 PMC8179238

[32] Mashimo T, Pichumani K, Vemireddy V, Hatanpaa KJ, Singh DK, Sirasanagandla S, et al. Acetate is a bioenergetic substrate for human glioblastoma and brain metastases. Cell. 2014;159:1603–14. 25525878 10.1016/j.cell.2014.11.025 PMC4374602

[33] Li X, Qian X, Lu Z. Local histone acetylation by ACSS2 promotes gene transcription for lysosomal biogenesis and autophagy. Autophagy. 2017;13:1790–1. 28820290 10.1080/15548627.2017.1349581 PMC5640193

[34] Li X, Yu W, Qian X, Xia Y, Zheng Y, Lee JH, et al. Nucleus-translocated ACSS2 promotes gene transcription for lysosomal biogenesis and autophagy. Mol Cell. 2017;66:684–97.e9. 28552616 10.1016/j.molcel.2017.04.026 PMC5521213

[35] Ciraku L, Bacigalupa ZA, Ju J, Moeller RA, Le Minh G, Lee RH, et al. O-GlcNAc transferase regulates glioblastoma acetate metabolism via regulation of CDK5-dependent ACSS2 phosphorylation. Oncogene. 2022;41:2122–36. 35190642 10.1038/s41388-022-02237-6 PMC9410282

[36] Esquea EM, Ciraku L, Young RG, Merzy J, Talarico AN, Ahmed NN, et al. Selective and brain-penetrant ACSS2 inhibitors target breast cancer brain metastatic cells. Front Pharmacol. 2024;15:1394685. 38818373 10.3389/fphar.2024.1394685 PMC11137182

[37] Gu D, Ye M, Zhu G, Bai J, Chen J, Yan L, et al. Hypoxia upregulating ACSS2 enhances lipid metabolism reprogramming through HMGCS1 mediated PI3K/AKT/mTOR pathway to promote the progression of pancreatic neuroendocrine neoplasms. J Transl Med. 2024;22:93. 38263056 10.1186/s12967-024-04870-z PMC10804556

[38] George Warren W, Osborn M, Yates A, O’Sullivan SE. The emerging role of fatty acid binding protein 7 (FABP7) in cancers. Drug Discov Today. 2024;29:103980. 38614160 10.1016/j.drudis.2024.103980

[39] Hoang-Minh LB, Siebzehnrubl FA, Yang C, Suzuki-Hatano S, Dajac K, Loche T, et al. Infiltrative and drug-resistant slow-cycling cells support metabolic heterogeneity in glioblastoma. EMBO J. 2018;37:e98772. 30322894 10.15252/embj.201798772 PMC6276884

[40] Bensaad K, Favaro E, Lewis CA, Peck B, Lord S, Collins JM, et al. Fatty acid uptake and lipid storage induced by HIF-1alpha contribute to cell growth and survival after hypoxia-reoxygenation. Cell Rep. 2014;9:349–65. 25263561 10.1016/j.celrep.2014.08.056

[41] De Rosa A, Pellegatta S, Rossi M, Tunici P, Magnoni L, Speranza MC, et al. A radial glia gene marker, fatty acid binding protein 7 (FABP7), is involved in proliferation and invasion of glioblastoma cells. PLoS ONE. 2012;7:e52113. 23284888 10.1371/journal.pone.0052113 PMC3528762

[42] Liang Y, Bollen AW, Aldape KD, Gupta N. Nuclear FABP7 immunoreactivity is preferentially expressed in infiltrative glioma and is associated with poor prognosis in EGFR-overexpressing glioblastoma. BMC Cancer. 2006;6:97. 16623952 10.1186/1471-2407-6-97 PMC1479358

[43] Kagawa Y, Umaru BA, Shima H, Ito R, Zama R, Islam A, et al. FABP7 regulates acetyl-CoA metabolism through the interaction with ACLY in the nucleus of astrocytes. Mol Neurobiol. 2020;57:4891–910. 32812201 10.1007/s12035-020-02057-3 PMC7541391

[44] Mita R, Beaulieu MJ, Field C, Godbout R. Brain fatty acid-binding protein and omega-3/omega-6 fatty acids: mechanistic insight into malignant glioma cell migration. J Biol Chem. 2010;285:37005–15. 20834042 10.1074/jbc.M110.170076 PMC2978629

[45] Choi WS, Xu X, Goruk S, Wang Y, Patel S, Chow M, et al. FABP7 facilitates uptake of docosahexaenoic acid in glioblastoma neural stem-like cells. Nutrients. 2021;13:2664. 34444824 10.3390/nu13082664 PMC8402214

[46] Umaru BA, Kagawa Y, Ohsaki Y, Pan Y, Chen CT, Chen DK, et al. Oleic acid‐bound FABP7 drives glioma cell proliferation through regulation of nuclear lipid droplet formation. FEBS J. 2022;290:1798–821. 36325660 10.1111/febs.16672

[47] Villa GR, Hulce JJ, Zanca C, Bi J, Ikegami S, Cahill GL, et al. An LXR-cholesterol axis creates a metabolic co-dependency for brain cancers. Cancer Cell. 2016;30:683–93. 27746144 10.1016/j.ccell.2016.09.008 PMC5479636

[48] Bjorkhem I, Meaney S. Brain cholesterol: long secret life behind a barrier. Arterioscler Thromb Vasc Biol. 2004;24:806–15. 14764421 10.1161/01.ATV.0000120374.59826.1b

[49] Bhat K, Saki M, Cheng F, He L, Zhang L, Ioannidis A, et al. Dopamine receptor antagonists, radiation, and cholesterol biosynthesis in mouse models of glioblastoma. J Natl Cancer Inst. 2021;113:1094–104. 33556960 10.1093/jnci/djab018 PMC8328983

[50] Hao M, Hou S, Li W, Li K, Xue L, Hu Q, et al. Combination of metabolic intervention and T cell therapy enhances solid tumor immunotherapy. Sci Transl Med. 2020;12:eaaz6667. 33239389 10.1126/scitranslmed.aaz6667

[51] Qiu Z, Yuan W, Chen T, Zhou C, Liu C, Huang Y, et al. HMGCR positively regulated the growth and migration of glioblastoma cells. Gene. 2016;576:22–7. 26432005 10.1016/j.gene.2015.09.067

[52] Wang X, Huang Z, Wu Q, Prager BC, Mack SC, Yang K, et al. MYC-regulated mevalonate metabolism maintains brain tumor-initiating cells. Cancer Res. 2017;77:4947–60. 28729418 10.1158/0008-5472.CAN-17-0114 PMC5600855

[53] Qiu R, Zhang J, Ge C, Zhong Y, Liu S, Li Q, et al. Ginsenosides Rg1 and CK control temozolomide resistance in glioblastoma cells by modulating cholesterol efflux and lipid raft distribution. Evid Based Complement Alternat Med. 2022;2022:1897508. 36276866 10.1155/2022/1897508 PMC9583863

[54] Irie N, Mizoguchi K, Warita T, Nakano M, Sasaki K, Tashiro J, et al. Repurposing of the cardiovascular drug statin for the treatment of cancers: efficacy of statin-dipyridamole combination treatment in melanoma cell lines. Biomedicines. 2024;12:698. 38540310 10.3390/biomedicines12030698 PMC10968169

[55] Jiang P, Mukthavaram R, Chao Y, Omura N, Bharati IS, Fogal V, et al. In vitro and in vivo anticancer effects of mevalonate pathway modulation on human cancer cells. Br J Cancer. 2014;111:1562–71. 25093497 10.1038/bjc.2014.431 PMC4200085

[56] Yanae M, Tsubaki M, Satou T, Itoh T, Imano M, Yamazoe Y, et al. Statin-induced apoptosis via the suppression of ERK1/2 and Akt activation by inhibition of the geranylgeranyl-pyrophosphate biosynthesis in glioblastoma. J Exp Clin Cancer Res. 2011;30:74. 21831290 10.1186/1756-9966-30-74 PMC3163617

[57] Bruinsmann FA, de Cristo Soares Alves A, de Fraga Dias A, Lopes Silva LF, Visioli F, Raffin Pohlmann A, et al. Nose-to-brain delivery of simvastatin mediated by chitosan-coated lipid-core nanocapsules allows for the treatment of glioblastoma in vivo. Int J Pharm. 2022;616:121563. 35151819 10.1016/j.ijpharm.2022.121563

[58] Rendon LF, Tewarie IA, Cote DJ, Gabriel A, Smith TR, Broekman MLD, et al. Statins and gliomas: a systematic review of the preclinical studies and meta-analysis of the clinical literature. Drugs. 2022;82:293–310. 35122635 10.1007/s40265-021-01668-x

[59] Cote DJ, Rosner BA, Smith-Warner SA, Egan KM, Stampfer MJ. Statin use, hyperlipidemia, and risk of glioma. Eur J Epidemiol. 2019;34:997–1011. 31559554 10.1007/s10654-019-00565-8 PMC7206659

[60] Mukhopadhyay TK, Willems S, Arp CJ, Morstein J, Haake CT, Merk D, et al. Development of light-activated LXR agonists. ChemMedChem. 2023;18:e202200647. 36896647 10.1002/cmdc.202200647

[61] Okabe A, Urano Y, Itoh S, Suda N, Kotani R, Nishimura Y, et al. Adaptive responses induced by 24S-hydroxycholesterol through Liver X receptor pathway reduce 7-ketocholesterol-caused neuronal cell death. Redox Biol. 2013;2:28–35. 24371802 10.1016/j.redox.2013.11.007 PMC3871289

[62] Cigliano L, Spagnuolo MS, Napolitano G, Iannotta L, Fasciolo G, Barone D, et al. 24S-hydroxycholesterol affects redox homeostasis in human glial U-87MG cells. Mol Cell Endocrinol. 2019;486:25–33. 30802527 10.1016/j.mce.2019.02.013

[63] Eibinger G, Fauler G, Bernhart E, Frank S, Hammer A, Wintersperger A, et al. On the role of 25-hydroxycholesterol synthesis by glioblastoma cell lines. Implications for chemotactic monocyte recruitment. Exp Cell Res. 2013;319:1828–38. 23541792 10.1016/j.yexcr.2013.03.025 PMC4052403

[64] Tricarico PM, Gratton R, Braga L, Celsi F, Crovella S. 25-Hydroxycholesterol and inflammation in Lovastatin-deregulated mevalonate pathway. Int J Biochem Cell Biol. 2017;92:26–33. 28918367 10.1016/j.biocel.2017.09.007

[65] Liu L, Li MY, Xing Y, Wang XY, Wang Y. The oncogenic roles of 27-hydroxycholesterol in glioblastoma. Oncol Lett. 2019;18:3623–9. 31579088 10.3892/ol.2019.10690 PMC6757262

[66] Liu W, Chakraborty B, Safi R, Kazmin D, Chang CY, McDonnell DP. Dysregulated cholesterol homeostasis results in resistance to ferroptosis increasing tumorigenicity and metastasis in cancer. Nat Commun. 2021;12:5103. 34429409 10.1038/s41467-021-25354-4 PMC8385107

[67] Yu J, Li P. The size matters: regulation of lipid storage by lipid droplet dynamics. Sci China Life Sci. 2017;60:46–56. 27981432 10.1007/s11427-016-0322-x

[68] Li Z, Liu H, Luo X. Lipid droplet and its implication in cancer progression. Am J Cancer Res. 2020;10:4112–22. 33414989 PMC7783747

[69] Geng F, Cheng X, Wu X, Yoo JY, Cheng C, Guo JY, et al. Inhibition of SOAT1 suppresses glioblastoma growth via blocking SREBP-1-mediated lipogenesis. Clin Cancer Res. 2016;22:5337–48. 27281560 10.1158/1078-0432.CCR-15-2973 PMC5093025

[70] Shakya S, Gromovsky AD, Hale JS, Knudsen AM, Prager B, Wallace LC, et al. Altered lipid metabolism marks glioblastoma stem and non-stem cells in separate tumor niches. Acta Neuropathol Commun. 2021;9:101. 34059134 10.1186/s40478-021-01205-7 PMC8166002

[71] Wu X, Geng F, Cheng X, Guo Q, Zhong Y, Cloughesy TF, et al. Lipid droplets maintain energy homeostasis and glioblastoma growth via autophagic release of stored fatty acids. iScience. 2020;23:101569. 33083736 10.1016/j.isci.2020.101569 PMC7549116

[72] Minami JK, Morrow D, Bayley NA, Fernandez EG, Salinas JJ, Tse C, et al. CDKN2A deletion remodels lipid metabolism to prime glioblastoma for ferroptosis. Cancer Cell. 2023;41:1048–60.e49. 37236196 10.1016/j.ccell.2023.05.001 PMC10330677

[73] Hsia JZ, Liu D, Haynes L, Cruz-Cosme R, Tang Q. Lipid droplets: formation, degradation, and their role in cellular responses to flavivirus infections. Microorganisms. 2024;12:647. 38674592 10.3390/microorganisms12040647 PMC11051834

[74] Liu R, Lee JH, Li J, Yu R, Tan L, Xia Y, et al. Choline kinase alpha 2 acts as a protein kinase to promote lipolysis of lipid droplets. Mol Cell. 2021;81:2722–35.e9. 34077757 10.1016/j.molcel.2021.05.005

[75] Ogasawara Y, Tsuji T, Fujimoto T. Multifarious roles of lipid droplets in autophagy—target, product, and what else? Semin Cell Dev Biol. 2020;108:47–54. 32169402 10.1016/j.semcdb.2020.02.013

[76] Ascenzi F, De Vitis C, Maugeri-Sacca M, Napoli C, Ciliberto G, Mancini R. SCD1, autophagy and cancer: implications for therapy. J Exp Clin Cancer Res. 2021;40:265. 34429143 10.1186/s13046-021-02067-6 PMC8383407

[77] Majuelos-Melguizo J, Rodriguez-Vargas JM, Martinez-Lopez N, Delgado-Bellido D, Garcia-Diaz A, Yuste VJ, et al.Glioblastoma cells counteract PARP inhibition through pro-survival induction of lipid droplets synthesis and utilization.Cancers. 2022;14:726. 35158994 10.3390/cancers14030726 PMC8833394

[78] Zhang I, Cui Y, Amiri A, Ding Y, Campbell RE, Maysinger D. Pharmacological inhibition of lipid droplet formation enhances the effectiveness of curcumin in glioblastoma. Eur J Pharm Biopharm. 2016;100:66–76. 26763536 10.1016/j.ejpb.2015.12.008

[79] Alkotub B, Bauer L, Bashiri Dezfouli A, Hachani K, Ntziachristos V, Multhoff G, et al. Radiosensitizing capacity of fenofibrate in glioblastoma cells depends on lipid metabolism. Redox Biol. 2025;79:103452. 39667305 10.1016/j.redox.2024.103452 PMC11697781

[80] Sun S, Qi G, Chen H, He D, Ma D, Bie Y, et al. Ferroptosis sensitization in glioma: exploring the regulatory mechanism of SOAT1 and its therapeutic implications. Cell Death Dis. 2023;14:754. 37980334 10.1038/s41419-023-06282-1 PMC10657441

[81] Lohr M, Hartig W, Schulze A, Kroiss M, Sbiera S, Lapa C, et al. SOAT1: a suitable target for therapy in high-grade astrocytic glioma? Int J Mol Sci. 2022;23:3726. 35409086 10.3390/ijms23073726 PMC8998855

[82] Yamanaka K, Urano Y, Takabe W, Saito Y, Noguchi N. Induction of apoptosis and necroptosis by 24(S)-hydroxycholesterol is dependent on activity of acyl-CoA:cholesterol acyltransferase 1. Cell Death Dis. 2014;5:e990. 24407243 10.1038/cddis.2013.524 PMC4040651

[83] Ayyagari VN, Wang X, Diaz-Sylvester PL, Groesch K, Brard L. Assessment of acyl-CoA cholesterol acyltransferase (ACAT-1) role in ovarian cancer progression—an in vitro study. PLoS ONE. 2020;15:e0228024. 31978092 10.1371/journal.pone.0228024 PMC6980601

[84] Bemlih S, Poirier MD, El Andaloussi A. Acyl-coenzyme A: cholesterol acyltransferase inhibitor Avasimibe affect survival and proliferation of glioma tumor cell lines. Cancer Biol Ther. 2010;9:1025–32. 20404512 10.4161/cbt.9.12.11875

[85] Liu JY, Fu WQ, Zheng XJ, Li W, Ren LW, Wang JH, et al. Avasimibe exerts anticancer effects on human glioblastoma cells via inducing cell apoptosis and cell cycle arrest. Acta Pharmacol Sin. 2021;42:97–107. 32451414 10.1038/s41401-020-0404-8 PMC7921416

[86] Sbiera S, Leich E, Liebisch G, Sbiera I, Schirbel A, Wiemer L, et al. Mitotane inhibits sterol-O-acyl transferase 1 triggering lipid-mediated endoplasmic reticulum stress and apoptosis in adrenocortical carcinoma cells. Endocrinology. 2015;156:3895–908. 26305886 10.1210/en.2015-1367

[87] Nguyen TB, Louie SM, Daniele JR, Tran Q, Dillin A, Zoncu R, et al. DGAT1-dependent lipid droplet biogenesis protects mitochondrial function during starvation-induced autophagy. Dev Cell. 2017;42:9–21.e25. 28697336 10.1016/j.devcel.2017.06.003 PMC5553613

[88] Kang H, Lee H, Kim K, Shin E, Kim B, Kang J, et al. DGKB mediates radioresistance by regulating DGAT1-dependent lipotoxicity in glioblastoma. Cell Rep Med. 2023;4:100800. 10.1016/j.xcrm.2022.100880 PMC9873821 36603576

[89] Cheng X, Geng F, Pan M, Wu X, Zhong Y, Wang C, et al. Targeting DGAT1 ameliorates glioblastoma by increasing fat catabolism and oxidative stress. Cell Metab. 2020;32:229–42.e8. 32559414 10.1016/j.cmet.2020.06.002 PMC7415721

[90] Dierge E, Debock E, Guilbaud C, Corbet C, Mignolet E, Mignard L, et al. Peroxidation of n-3 and n-6 polyunsaturated fatty acids in the acidic tumor environment leads to ferroptosis-mediated anticancer effects. Cell Metab. 2021;33:1701–15.e5. 34118189 10.1016/j.cmet.2021.05.016

[91] Zhang Y, Zhang XY, Shi SR, Ma CN, Lin YP, Song WG, et al. Natural products in atherosclerosis therapy by targeting PPARs: a review focusing on lipid metabolism and inflammation. Front Cardiovasc Med. 2024;11:1372055. 38699583 10.3389/fcvm.2024.1372055 PMC11064802

[92] Zeng W, Yin X, Jiang Y, Jin L, Liang W. PPARalpha at the crossroad of metabolic-immune regulation in cancer. FEBS J. 2022;289:7726–39. 34480827 10.1111/febs.16181

[93] Li Y, Pan Y, Zhao X, Wu S, Li F, Wang Y, et al. Peroxisome proliferator-activated receptors: a key link between lipid metabolism and cancer progression. Clin Nutr. 2024;43:332–45. 38142478 10.1016/j.clnu.2023.12.005

[94] Courtney R, Landreth GE. LXR regulation of brain cholesterol: from development to disease. Trends Endocrinol Metab. 2016;27:404–14. 27113081 10.1016/j.tem.2016.03.018 PMC4986614

[95] Pirmoradi L, Seyfizadeh N, Ghavami S, Zeki AA, Shojaei S. Targeting cholesterol metabolism in glioblastoma: a new therapeutic approach in cancer therapy. J Investig Med. 2019;67:715–9. 30765502 10.1136/jim-2018-000962

[96] Fan Y, Zhang R, Wang C, Pan M, Geng F, Zhong Y, et al. STAT3 activation of SCAP-SREBP-1 signaling upregulates fatty acid synthesis to promote tumor growth. J Biol Chem. 2024;300:107351. 38718868 10.1016/j.jbc.2024.107351 PMC11176798

[97] Geng F, Zhong Y, Su H, Lefai E, Magaki S, Cloughesy TF, et al. SREBP-1 upregulates lipophagy to maintain cholesterol homeostasis in brain tumor cells. Cell Rep. 2023;42:112790. 37436895 10.1016/j.celrep.2023.112790 PMC10528745

[98] Xu S, Smothers JC, Rye D, Endapally S, Chen H, Li S, et al. A cholesterol-binding bacterial toxin provides a strategy for identifying a specific Scap inhibitor that blocks lipid synthesis in animal cells. Proc Natl Acad Sci USA. 2024;121:e2318024121. 38330014 10.1073/pnas.2318024121 PMC10873635

[99] Chakravarthy MV, Zhu Y, Lopez M, Yin L, Wozniak DF, Coleman T, et al. Brain fatty acid synthase activates PPARalpha to maintain energy homeostasis. J Clin Investig. 2007;117:2539–52. 17694178 10.1172/JCI31183 PMC1937501

[100] Benedetti E, d’Angelo M, Ammazzalorso A, Gravina GL, Laezza C, Antonosante A, et al. PPARalpha antagonist AA452 triggers metabolic reprogramming and increases sensitivity to radiation therapy in human glioblastoma primary cells. J Cell Physiol. 2017;232:1458–66. 27736000 10.1002/jcp.25648

[101] Venkatesh D, O’Brien NA, Zandkarimi F, Tong DR, Stokes ME, Dunn DE, et al. MDM2 and MDMX promote ferroptosis by PPARalpha-mediated lipid remodeling. Genes Dev. 2020;34:526–43. 32079652 10.1101/gad.334219.119 PMC7111265

[102] Han DF, Zhang JX, Wei WJ, Tao T, Hu Q, Wang YY, et al. Fenofibrate induces G0/G1 phase arrest by modulating the PPARalpha/FoxO1/p27 kip pathway in human glioblastoma cells. Tumour Biol. 2015;36:3823–9. 25566967 10.1007/s13277-014-3024-4

[103] Haynes HR, White P, Hares KM, Redondo J, Kemp KC, Singleton WGB, et al. The transcription factor PPARalpha is overexpressed and is associated with a favourable prognosis in IDH-wildtype primary glioblastoma. Histopathology. 2017;70:1030–43. 27926792 10.1111/his.13142

[104] Montaigne D, Butruille L, Staels B. PPAR control of metabolism and cardiovascular functions. Nat Rev Cardiol. 2021;18:809–23. 34127848 10.1038/s41569-021-00569-6

[105] Hua TNM, Oh J, Kim S, Antonio JM, Vo VTA, Om J, et al. Peroxisome proliferator-activated receptor gamma as a theragnostic target for mesenchymal-type glioblastoma patients. Exp Mol Med. 2020;52:629–42. 32280134 10.1038/s12276-020-0413-1 PMC7210935

[106] Cipolletta D, Feuerer M, Li A, Kamei N, Lee J, Shoelson SE, et al. PPAR-gamma is a major driver of the accumulation and phenotype of adipose tissue Treg cells. Nature. 2012;486:549–53. 22722857 10.1038/nature11132 PMC3387339

[107] Angela M, Endo Y, Asou HK, Yamamoto T, Tumes DJ, Tokuyama H, et al. Fatty acid metabolic reprogramming via mTOR-mediated inductions of PPARgamma directs early activation of T cells. Nat Commun. 2016;7:13683. 27901044 10.1038/ncomms13683 PMC5141517

[108] Ercolano G, Gomez-Cadena A, Dumauthioz N, Vanoni G, Kreutzfeldt M, Wyss T, et al. PPARɣ drives IL-33-dependent ILC2 pro-tumoral functions. Nat Commun. 2021;12:2538. 33953160 10.1038/s41467-021-22764-2 PMC8100153

[109] Nguyen TTT, Ishida CT, Shang E, Shu C, Torrini C, Zhang Y, et al. Activation of LXRbeta inhibits tumor respiration and is synthetically lethal with Bcl-xL inhibition. EMBO Mol Med. 2019;11:e10769. 31468706 10.15252/emmm.201910769 PMC6783693

[110] Guo D, Reinitz F, Youssef M, Hong C, Nathanson D, Akhavan D, et al. An LXR agonist promotes glioblastoma cell death through inhibition of an EGFR/AKT/SREBP-1/LDLR-dependent pathway. Cancer Discov. 2011;1:442–56. 22059152 10.1158/2159-8290.CD-11-0102 PMC3207317

[111] Tavazoie MF, Pollack I, Tanqueco R, Ostendorf BN, Reis BS, Gonsalves FC, et al. LXR/ApoE activation restricts innate immune suppression in cancer. Cell. 2018;172:825–40.e18. 29336888 10.1016/j.cell.2017.12.026 PMC5846344

[112] Patel D, Ahmad F, Kambach DM, Sun Q, Halim AS, Kramp T, et al. LXRbeta controls glioblastoma cell growth, lipid balance, and immune modulation independently of ABCA1. Sci Rep. 2019;9:15458. 31664073 10.1038/s41598-019-51865-8 PMC6820787

[113] Geng F, Guo D. SREBF1/SREBP-1 concurrently regulates lipid synthesis and lipophagy to maintain lipid homeostasis and tumor growth. Autophagy. 2024;20:1183–5. 37927089 10.1080/15548627.2023.2275501 PMC11135807

[114] Chandrasekaran P, Weiskirchen R. The role of SCAP/SREBP as central regulators of lipid metabolism in hepatic steatosis. Int J Mol Sci. 2024;25:1109. 38256181 10.3390/ijms25021109 PMC10815951

[115] Shimano H, Sato R. SREBP-regulated lipid metabolism: convergent physiology—divergent pathophysiology. Nat Rev Endocrinol. 2017;13:710–30. 28849786 10.1038/nrendo.2017.91

[116] Cheng C, Ru P, Geng F, Liu J, Yoo JY, Wu X, et al. Glucose-mediated N-glycosylation of SCAP is essential for SREBP-1 activation and tumor growth. Cancer Cell. 2015;28:569–81. 26555173 10.1016/j.ccell.2015.09.021 PMC4643405

[117] Guo D, Prins RM, Dang J, Kuga D, Iwanami A, Soto H, et al. EGFR signaling through an Akt-SREBP-1-dependent, rapamycin-resistant pathway sensitizes glioblastomas to antilipogenic therapy. Sci Signal. 2009;2:ra82. 20009104 10.1126/scisignal.2000446 PMC2978002

[118] Lim SA, Wei J, Nguyen TM, Shi H, Su W, Palacios G, et al. Lipid signalling enforces functional specialization of T(reg) cells in tumours. Nature. 2021;591:306–11. 33627871 10.1038/s41586-021-03235-6 PMC8168716

[119] Choi WI, Jeon BN, Park H, Yoo JY, Kim YS, Koh DI, et al. Proto-oncogene FBI-1 (Pokemon) and SREBP-1 synergistically activate transcription of fatty-acid synthase gene (FASN). J Biol Chem. 2008;283:29341–54. 18682402 10.1074/jbc.M802477200 PMC2662021

[120] Gholkar AA, Cheung K, Williams KJ, Lo YC, Hamideh SA, Nnebe C, et al. Fatostatin inhibits cancer cell proliferation by affecting mitotic microtubule spindle assembly and cell division. J Biol Chem. 2016;291:17001–8. 27378817 10.1074/jbc.C116.737346 PMC5016105

[121] Williams KJ, Argus JP, Zhu Y, Wilks MQ, Marbois BN, York AG, et al. An essential requirement for the SCAP/SREBP signaling axis to protect cancer cells from lipotoxicity. Cancer Res. 2013;73:2850–62. 23440422 10.1158/0008-5472.CAN-13-0382-T PMC3919498

[122] Lamoral-Theys D, Andolfi A, Van Goietsenoven G, Cimmino A, Le Calve B, Wauthoz N, et al. Lycorine, the main phenanthridine Amaryllidaceae alkaloid, exhibits significant antitumor activity in cancer cells that display resistance to proapoptotic stimuli: an investigation of structure-activity relationship and mechanistic insight. J Med Chem. 2009;52:6244–56. 19788245 10.1021/jm901031h PMC3205972

[123] Zheng ZG, Zhu ST, Cheng HM, Zhang X, Cheng G, Thu PM, et al. Discovery of a potent SCAP degrader that ameliorates HFD-induced obesity, hyperlipidemia and insulin resistance via an autophagy-independent lysosomal pathway. Autophagy. 2021;17:1592–613. 32432943 10.1080/15548627.2020.1757955 PMC8354609

[124] Liu Y, Hua W, Li Y, Xian X, Zhao Z, Liu C, et al. Berberine suppresses colon cancer cell proliferation by inhibiting the SCAP/SREBP-1 signaling pathway-mediated lipogenesis. Biochem Pharmacol. 2020;174:113776. 31874145 10.1016/j.bcp.2019.113776

[125] Wang J, Qi Q, Feng Z, Zhang X, Huang B, Chen A, et al. Berberine induces autophagy in glioblastoma by targeting the AMPK/mTOR/ULK1-pathway. Oncotarget. 2016;7:66944–58. 27557493 10.18632/oncotarget.11396 PMC5341849

[126] Comincini S, Manai F, Sorrenti M, Perteghella S, D'Amato C, Miele D, et al. Development of berberine-loaded nanoparticles for astrocytoma cells administration and photodynamic therapy stimulation. Pharmaceutics. 2023;15:1078. 37111564 10.3390/pharmaceutics15041078 PMC10146331

[127] Facchini G, Ignarro RS, Rodrigues-Silva E, Vieira AS, Lopes-Cendes I, Castilho RF, et al. Toxic effects of phytol and retinol on human glioblastoma cells are associated with modulation of cholesterol and fatty acid biosynthetic pathways. J Neuro Oncol. 2017;136:435–43. 10.1007/s11060-017-2672-9 29159775

[128] Camargo N, Goudriaan A, van Deijk A-LF, Otte WM, Brouwers JF, Lodder H, et al. Oligodendroglial myelination requires astrocyte-derived lipids. PLOS Biol. 2017;15:e1002605. 28549068 10.1371/journal.pbio.1002605 PMC5446120

[129] Dai G, Huang S, Li Y, Tu X, Xia J, Zhou Z, et al. Mettl3-mediated m(6)A modification plays a role in lipid metabolism disorders and progressive liver damage in mice by regulating lipid metabolism-related gene expression. Aging. 2023;15:5550–68. 37335109 10.18632/aging.204810 PMC10333091

[130] Gao H, Cheng X, Chen J, Ji C, Guo H, Qu W, et al. Fto-modulated lipid niche regulates adult neurogenesis through modulating adenosine metabolism. Hum Mol Genet. 2020;29:2775–87. 32766784 10.1093/hmg/ddaa171

[131] Song K, Sun H, Tu B, Zhou Y, Lin L-C, Liu Z-Y, et al. WTAP boosts lipid oxidation and induces diabetic cardiac fibrosis by enhancing AR methylation. iScience. 2023;26:107931. 37810250 10.1016/j.isci.2023.107931 PMC10558737

[132] Zhang S, Zhang P, Wu A, Xu Z, Huang S, Liu X, et al. Downregulated M6A modification and expression of circRNA_103239 promoted the progression of glioma by regulating the miR-182-5p/MTSS1 signalling pathway. J Cancer. 2023;14:3508–20. 38021156 10.7150/jca.85320 PMC10647192

[133] Zhang J, Liu B, Xu C, Ji C, Yin A, Liu Y, et al. Cholesterol homeostasis confers glioma malignancy triggered by hnRNPA2B1-dependent regulation of SREBP2 and LDLR. Neuro Oncol. 2024;26:684–700. 38070488 10.1093/neuonc/noad233 PMC10995519

[134] Deng X, Sun X, Hu Z, Wu Y, Zhou C, Sun J, et al. Exploring the role of m6A methylation regulators in glioblastoma multiforme and their impact on the tumor immune microenvironment. FASEB J. 2023;37:e23155. 37606566 10.1096/fj.202301343

[135] Ji Q, Guo Y, Li Z, Zhang X. WTAP regulates the production of reactive oxygen species, promotes malignant progression, and is closely related to the tumor microenvironment in glioblastoma. Aging. 2024;16:5601–17. 38535989 10.18632/aging.205666 PMC11006471

[136] Fan Y, Yan D, Ma L, Liu X, Luo G, Hu Y, et al. ALKBH5 is a prognostic factor and promotes the angiogenesis of glioblastoma. Sci Rep. 2024;14:1303. 38221546 10.1038/s41598-024-51994-9 PMC10788339

[137] Lv D, Zhong C, Dixit D, Yang K, Wu Q, Godugu B, et al. EGFR promotes ALKBH5 nuclear retention to attenuate N6-methyladenosine and protect against ferroptosis in glioblastoma. Mol Cell. 2023;83:4334–51.e37. 37979586 10.1016/j.molcel.2023.10.025 PMC10842222

[138] Darwish A, Pammer M, Gallyas F, Jr, Vígh L, Balogi Z, Juhász K. Emerging lipid targets in glioblastoma. Cancers. 2024;16:397. 38254886 10.3390/cancers16020397 PMC10814456

[139] Yu N, Aboud O. The lipidomic signature of glioblastoma: a promising frontier in cancer research. Cancers. 2024;16:1089. 38539424 10.3390/cancers16061089 PMC10968728

[140] Miska J, Chandel NS. Targeting fatty acid metabolism in glioblastoma. J Clin Investig. 2023;133:e163448. 36594473 10.1172/JCI163448 PMC9797338

[141] Kou Y, Geng F, Guo D. Lipid metabolism in glioblastoma: from de novo synthesis to storage. Biomedicines. 2022;10:1943. 36009491 10.3390/biomedicines10081943 PMC9405736

[142] Nazaruk E, Gajda E, Ziędalska I, Godlewska M, Gawel D. Enhancement of temozolomide stability and anticancer efficacy by loading in monopalmitolein-based cubic phase nanoparticles. ACS Omega. 2024;9:38936–45. 39310207 10.1021/acsomega.4c05291 PMC11411539

[143] Basso J, Fortuna A, Vitorino R, Vitorino C. Old drugs, new tricks: delivering pitavastatin-loaded nanostructured lipid carriers for glioblastoma treatment. Colloids Surf B Biointerfaces. 2025;245:114253. 39303387 10.1016/j.colsurfb.2024.114253

